# Single-molecule long-read sequencing analysis improves genome annotation and sheds new light on the transcripts and splice isoforms of *Zoysia japonica*

**DOI:** 10.1186/s12870-022-03640-7

**Published:** 2022-05-26

**Authors:** Jin Guan, Shuxia Yin, Yuesen Yue, Lingyun Liu, Yidi Guo, Hui Zhang, Xifeng Fan, Ke Teng

**Affiliations:** 1 Institute of Grassland, Flowers, and Ecology, Beijing Academy of Agriculture and Forestry Sciences, Beijing, 100097 China; 2 School of Grassland Science, Beijing Forestry University, Beijing, 100083 China

**Keywords:** *Zoysia japonica*, Full-length transcriptome, Alternative splicing, Fusion transcripts, Transcription factors, Senescence

## Abstract

**Background:**

*Zoysia japonica* is an important warm-season turfgrass used worldwide. Although the draft genome sequence and a vast amount of next-generation sequencing data have been published, the current genome annotation and complete mRNA structure remain incomplete. Therefore, to analyze the full-length transcriptome of *Z. japonica*, we used the PacBio single-molecule long-read sequencing method in this study.

**Results:**

First, we generated 37,056 high-confidence non-redundant transcripts from 16,005 gene loci. Next, 32,948 novel transcripts, 913 novel gene loci, 8035 transcription factors, 89 long non-coding RNAs, and 254 fusion transcripts were identified. Furthermore, 15,675 alternative splicing events and 5325 alternative polyadenylation sites were detected. In addition, using bioinformatics analysis, the underlying transcriptional mechanism of senescence was explored based on the revised reference transcriptome.

**Conclusion:**

This study provides a full-length reference transcriptome of *Z. japonica* using PacBio single-molecule long-read sequencing for the first time. These results contribute to our knowledge of the transcriptome and improve the knowledge of the reference genome of *Z. japonica*. This will also facilitate genetic engineering projects using *Z. japonica*.

**Supplementary Information:**

The online version contains supplementary material available at 10.1186/s12870-022-03640-7.

## Background

Structural and functional genomics research is the basis for understanding plant biology [1]. Genomic research contributes to a better understanding of the biological mechanisms, exploration and use of the best traits, and the design of more effective breeding strategies [2]. Moreover, the continuous update of genome sequences with transcriptome data will increase their use. Therefore, it is essential to obtain high-quality genome and transcriptome sequences.

Commonly known as a warm-season turfgrass, *Zoysia japonica* (2n = 4x = 40) has many remarkable characteristics, including minimal maintenance, excellent tolerance to drought, salinity, and freezing, good ability to conserve water and soil, and excellent traffic tolerance [3–8]. Nevertheless, the short green period and unaesthetic appearance during senescence hamper its further popularization and utilization [9, 10]. Owing to the relatively high rate of gene introgression and hybridization in *Z. japonica* [4, 6, 11], the genome sequence is of great help in the research on the structure and functional genomics of this species *Z. japonica*. The genome sequencing of *Z. japonica* was accomplished using the shotgun sequencing strategy and published in 2016 [11]. However, total mRNA sequences and structural characteristics are still largely unknown, especially in the absence of alternative splicing events (AS), long non-coding RNAs (lncRNAs), alternative polyadenylation (APA) sites, and fusion transcript information. Consequently, a large number of unannotated transcripts were overlooked. It is still necessary to perform full-length transcriptomic sequencing studies of *Z. japonica*.

Transcriptome research is an essential tool for understanding life processes. Next-generation high-throughput sequencing technology, a revolutionary tool to reduce the cost of high-throughput sequencing, helps us understand the expression levels and regulatory mechanisms of different genes better. The Illumina RNA-seq data has a much higher sequencing depth and significantly lower error rate, leading to much higher genome coverage. In recent years, short-read sequencing data of *Z. japonica* have accumulated [9, 12–14]. Studies have shown that plants have more complex processes, such as pre-transcriptional regulation, which mainly includes AS and alternative polyadenylation [15, 16], and is crucial for a deeper understanding of plant transcriptomes and their potential biological consequences. However, the Illumina RNA-seq data do not allow frequent and accurate collection or assembly of complete transcripts or identify certain information, such as splice isoforms or homologous genes. PacBio single-molecule real-time (SMRT) technology reveals full-length transcripts and provides highly confident transcript start and end sites. The ultra-long reads (median 10 kb) of this platform contain the sequence information of a single complete transcript, and post-analysis does not require assembly [17–19]. The emergence of third-generation sequencing has effectively made up for the shortcomings of second-generation sequencing. The emergence of third-generation sequencing has made it possible to analyze the full-length mRNA sequences of genes, accurately distinguish different splice isoforms, and identify APA sites, effectively compensating up for the deficiencies of second-generation sequencing.

Third-generation sequencing has been applied to many turfgrass and ground cover plants in our previous studies. Using the Pacific Bioscience Sequel System, 5492 AS events, 4333 lncRNA and 3762 fusion transcripts were identified in *Trifolium pratense*, effectively improving its genome annotation [20]. Using the Pacific Bioscience RS II platform, 58,328 annotated full-length transcripts and 5052 AS events consisting of seven alternative splicing types were identified for *Carex breviculmis*, providing an informative reference transcriptome [21]. The Pacific Bioscience RS II platform was also used to analyze the transcriptome of *Lolium perenne*. The sequencing data revealed 6709 AS events and 23,789 APA sites, improving the current genomic annotation of perennial ryegrass [22]. These studies provide useful information for further research on the transcriptome.

Here, we conducted PacBio single-molecule long-read sequencing analysis to explore the features of the full-length transcriptome and to improve the transcript annotation of the reference genome of *Z. japonica*. Based on the newly generated transcriptome and revised genome atlas, the underlying transcriptional mechanism of *Z. japonica* senescence was investigated through differentially expressed gene (DEG) analysis screened using the Illumina sequencing data. We expect this study to effectively improve the annotation of the genome sequence and provides useful information on the transcriptome of *Z. japonica*. We believe it will also provide valuable resources for further research on senescence studies in *Z. japonica*.

## Results

### Physiological changes during Z. *japonica* senescence

The physiological results are shown in Table S1. Throughout *Z. japonica* senescence, the chlorophyll content decreased significantly (*p* ≤ 0.05). At the same time, besides the weakened photosynthetic capacity with leafage, the photosynthetic rate (Pn), stomatal conductance (Gs), and transpiration rate (Tr) were significantly reduced (*p* ≤ 0.05), and the intercellular space CO_2_ concentration (Ci) increased significantly (*p* ≤ 0.05). Plant hormone content also underwent significant changes (*p* ≤ 0.05), such as a decrease in indole-3-acetic acid (IAA) and an increase in abscisic acid (ABA) contents. Changes in antioxidant enzyme activity enhanced the ability to remove reactive oxygen species. In addition, the relative water content of leaves at different developmental stages showed little difference, and the soluble sugar content and electrolyte leakage (EL) increased, which indicates that osmotic stress is induced in response to senescence. We used principal component analysis (PCA) to classify nine samples based on the physiological data determined (Fig. S1). The green circles represent young leaves, whereas the red and yellow circles represent mature and senescent leaves, respectively. The PCA results generated three different categories, which confirmed the reliability of the sequencing sample grouping (Fig. S1).

### Zoysiagrass transcriptome through SMRT sequencing

**Fig. 1:**
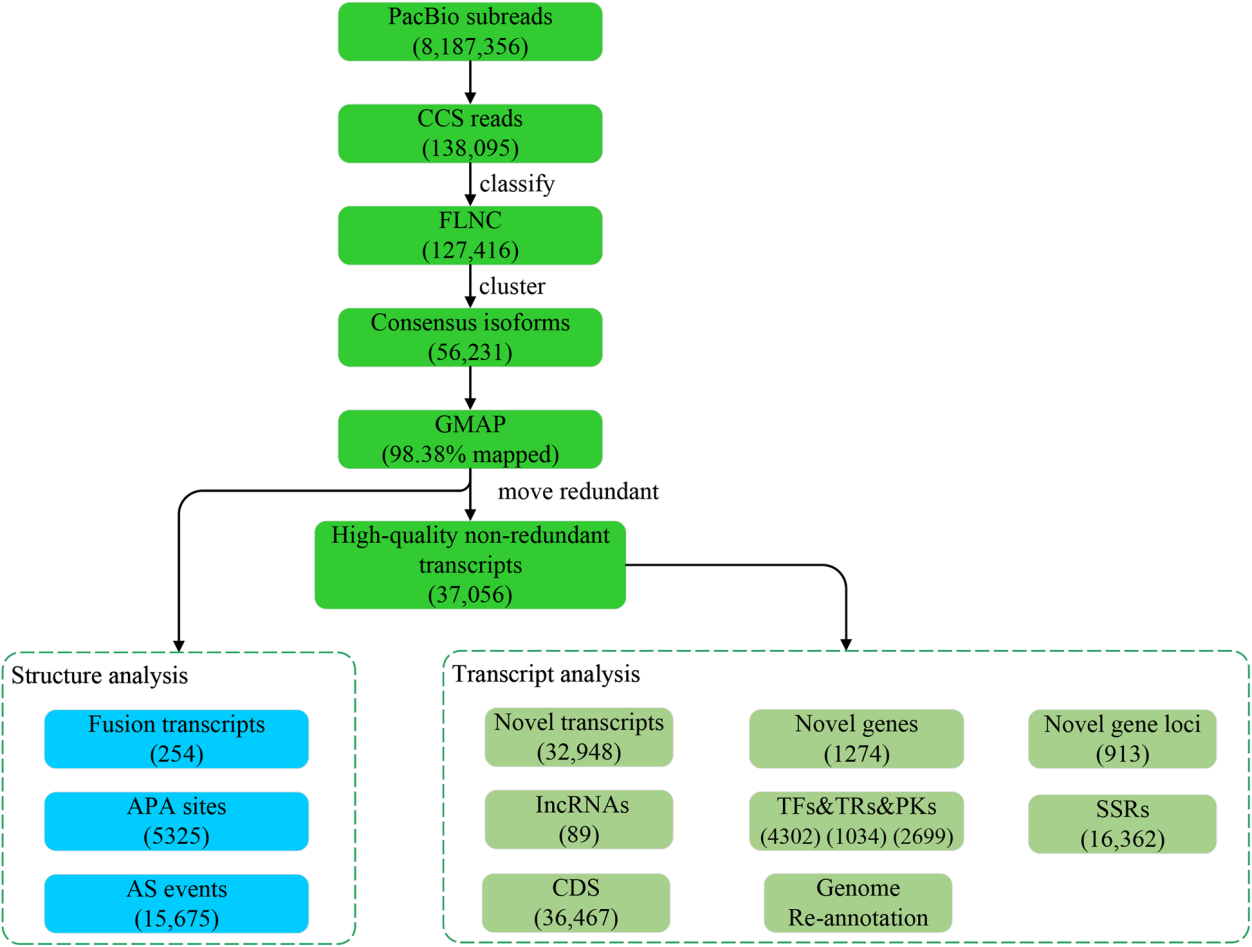
Flow chart of bioinformation analysis

To identify as many transcripts as possible, the total RNA was extracted from six organs of *Z. japonica* for sequencing and analysis with the PacBio Sequel II platform to capture full-length sequences accurately. The read length of the cDNA library was 1-6 kb, and we obtained 8,187,356 subreads (Fig. 1). To increase the use of the sequencing data, a new protocol was used to generate circular consensus (CCS) reads with full passes of ≥ 3 and predicted consensus accuracy of > 0.9. A total of 138,095 CCS reads were generated, and the mean number of passes was 54. The read length distribution of the CCS from the library is shown in Fig. S2A, and the mean CCS read length was 2563 base pairs (bp). Among full-length CCS sequences, 127,416 were identified as full-length non-chimeric (FLNC) sequences and the percentage of FLNC sequences in all CCS reads was 92.27%. The read length distribution of the FLNC sequences is shown in Fig. S2B. A total of 56,231 consensus sequences were clustered using the FLNC sequences, and 56,228 high-quality transcripts (accuracy > 0.9) were determined. The average consensus isoform read length was 2478 bp, and the length distribution of the consensus sequence is shown in Fig. S2C. The statistical information of sequencing data through SMRT is shown in Table 1.

**Table 1:** Statistics of sequencing data by SMRT

Category	Subread	CCS	FLNC	consensus isoforms
number	8,187,356	138,095	127,416	56,231
Min length/bp	51	207	56	56
Max length/bp	204,737	20,035	14,330	13,428
mean length/bp	2172	2,563	2458	2.478
N50 length/bp	2,444	2,863	2767	2,856

### Genome mapping

In the *Z. japonica* genome, 59,271 genes and transcripts were annotated; 56,231 (88.17%) transcripts contained less than eight exons, whereas 7010 (11.83%) transcripts contained more than seven exons and 230 (0.39%) transcripts contained more than 20 exons. In addition, 11,519 (19.43%) transcripts had no introns, 47,752 (80.57%) transcripts contained introns, and only 199 (0.34%) transcripts contained more than 20 introns.

**Fig. 2:**
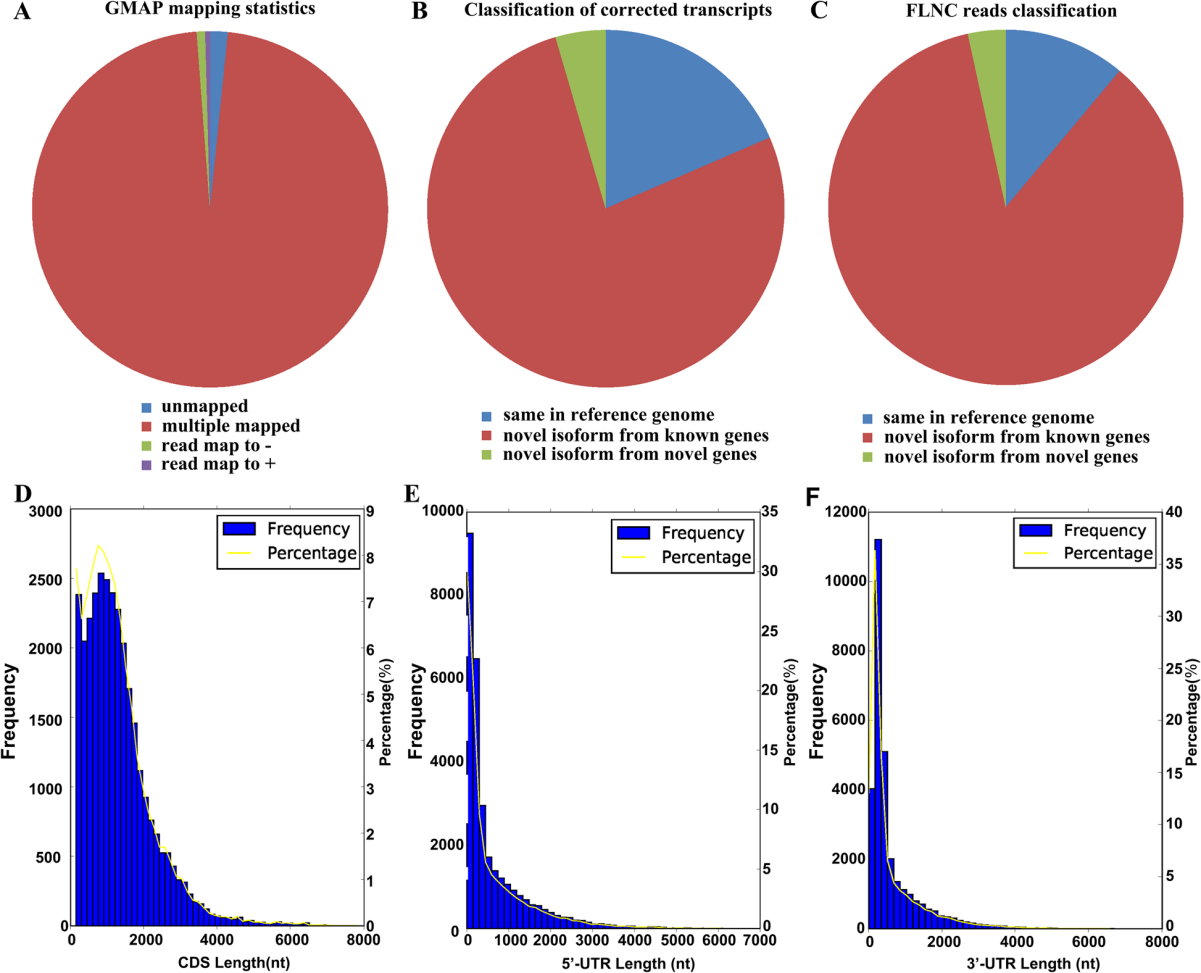
GMAP analysis and CDS-UTR structures of SMRT sequences. **A** GMAP analysis of consensus isoforms. **B** Classification of FLNC sequences. **C** Classification of non-redundant transcripts. **D** Number, percentage and length distributions of coding sequences of de-redundant transcripts. Number, percentage and length distributions of 5’-UTR (**E**) and 3’-UTR (**F**) of non-redundant transcripts

A total of 56,231 consensus isoforms were compared with the transcript annotations of the reference genome using GMAP, and 55,320 (98.38%) reads were mapped successfully. Based on the results (Fig. 2A), these consensus isoforms were divided into four groups as follows: 54,649 reads (98.79%) showing multiple alignments to the genome (multiple mapped), 911 reads were unmapped reference genomes, 432 reads were mapped to the opposite strand of the genome (reads mapped to -), and 239 reads were mapped to the positive strand of the genome (reads mapped to +).

Using GMAP, we compared all FLNC sequences against the reference genome sequence. A total of 126,034 reads (98.92%) were mapped, including 23,333 isoforms that were the same as the reference genome, 96,975 novel isoforms from known genes and 5276 novel isoforms from novel genes (Fig. 2B). We obtained 37,056 high-quality non-redundant transcripts using the cDNA Cupcake software. We aligned 37,056 high-quality non-redundant transcripts with the genome to identify novel transcripts. The results showed that 4108 isoforms were the same as the reference genome, 31,674 novel isoforms were of known genes and 1274 novel isoforms were of novel genes (Fig. 2C). In total, 1274 novel genes, 32,948 novel transcripts and 913 novel gene loci were identified.

**Fig. 3:**
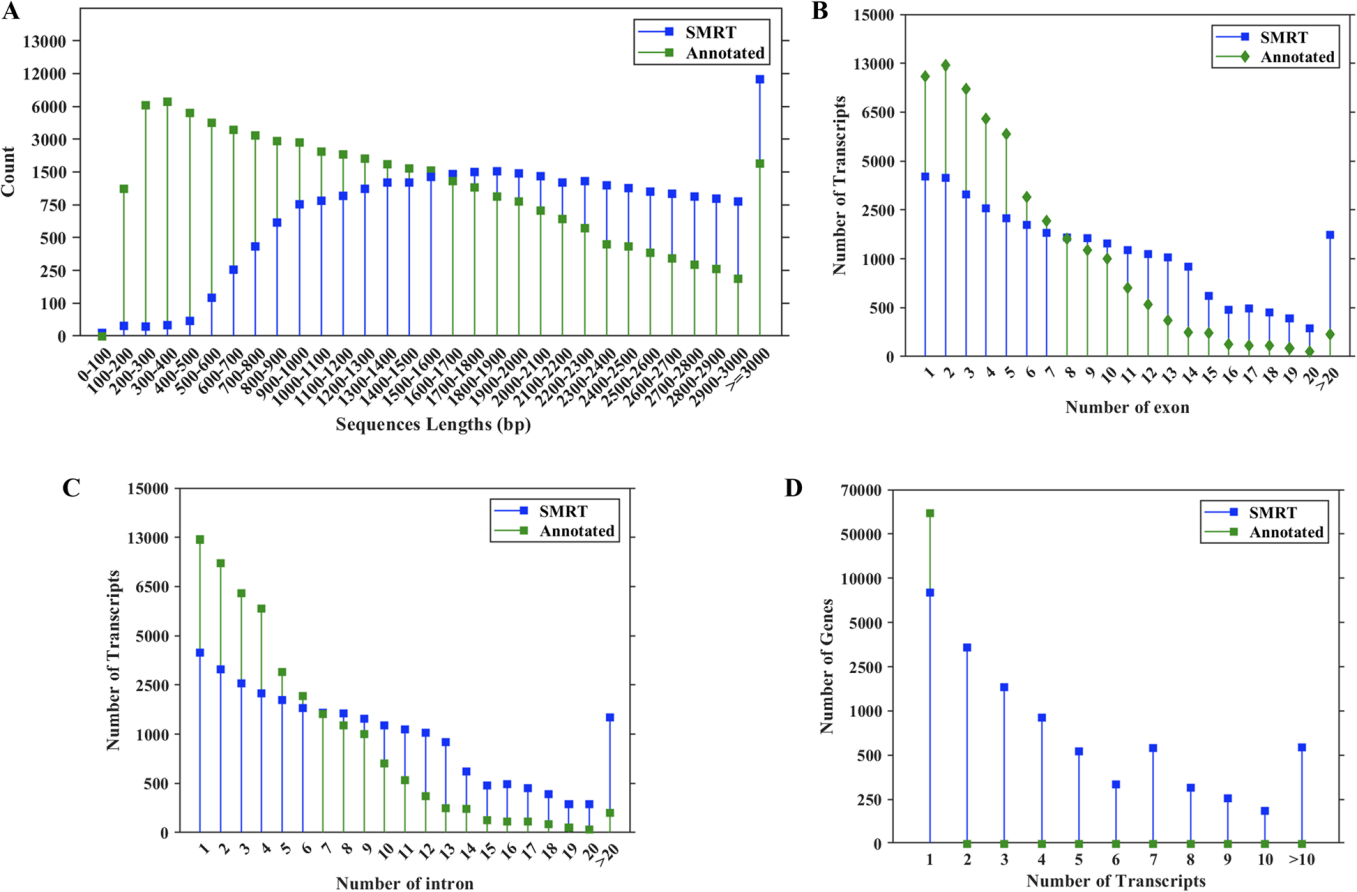
Comparison of sequencing data between SMRT and reference genome. **A** Comparison of transcript number and length distributions. **B** Number distribution of exons in transcripts. **C** Number distribution of introns in transcripts. **D** Distribution of genes that produce one or more splice isoforms

We compared length distribution and number between the transcripts identified from SMRT sequencing data and the annotated transcripts in the reference genome. We found that 59,271 transcripts annotated in the genome did not represent full-length cDNAs. A total of 62.36% of the transcripts from the reference genome were < 1000 bp, which was only 6.34% using SMRT sequencing data. Longer transcripts were identified through SMRT sequencing data rather than the annotated transcripts, and 29.50% of the transcripts from SMRT sequencing data were longer than 3000 bp (Fig. 3A).

### Coding region sequences (CDS) identification and exon–intron structure analysis

Reliable CDS were identified from non-redundant transcripts using TransDecoder software. A total of 36,467 CDS with a mean length of 1325.71 nucleotides were found, and 30,889 carried complete ORFs (Fig. 2D, Table 2). Transcripts consisting of 700–1000 amino acids were the most abundant, accounting for 21.13%. The protein sequence encoded by the complete ORF region is shown in Fig. 3B. In addition, the number, length distribution, and frequency of 5′ and 3′-UTRs were investigated. The results showed 4741 5′-UTRs with a mean length of 656.31 nucleotides and 746 3′-UTRs with a mean length of 674.54 nucleotides (Fig. 2E-F, Table 2).

**Table 2:** CDS identification from PacBio SMRT

	CDS	ORF	5'-UTR	3'-UTR	uncertain
number	36,467	30,889	746	4741	91

The exon and intron structures of 37,056 transcripts were analyzed: 22,478 transcripts (60.66%) had less than eight exons, 14,578 (39.34%) had more than seven exons, and 1977 (5.33%) had more than 20 exons (Fig. 3B). In addition, we predicted 4365 transcripts (11.78%) had no introns, 32,691 (88.22%) had introns, and 1689 (4.56%) had more than 20 introns (Fig. 3C). In the *Z. japonica* genome, 11,519 (31.09%) transcripts contained one exon, 12,767 (34.45%) transcripts contained two exons, 230 (0.39%) transcripts contained more than 20 exons (Fig. 3B), and only 0.12% of genes (199) had more than 20 introns (Fig. 3C).

### Fusion transcript, novel gene, and transcript validation

**Fig. 4:**
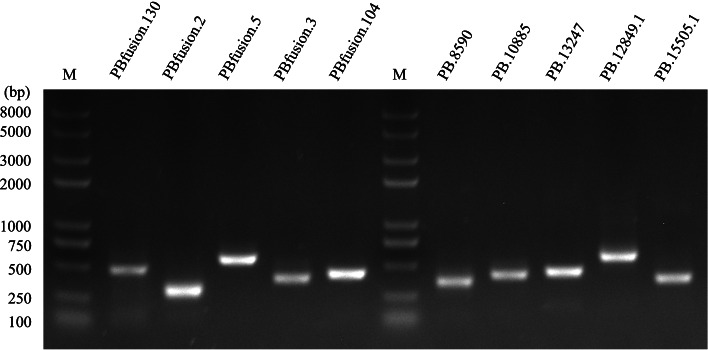
RT-PCR verification of fusion transcripts and novel genes

We identified 254 fusion transcripts using PacBio SMRT sequencing data and displayed the structural information of exons and introns in the fusion transcript (Table S2). The position of the fusion transcripts, and corresponding position of each exon on the transcript are shown in Table S2. To further validate the novel genes and fusion transcripts, five novel genes and five fusion transcripts were randomly selected for real-time PCR (RT-PCR) verification (Table S3) and Sanger sequencing. Primers were designed based on the nucleotide sequences provided by the SMRT sequencing data. Therefore, we amplified the DNA fragments successfully, and the size was consistent with SMRT sequencing data (Fig. 4). Then, we used Sanger sequencing to verify the sequences. In total, five genes and five fusion transcripts were experimentally validated. The results above demonstrated that SMRT sequencing technology was a reliable method for discovering novel genes and fusion transcripts. Functional annotation of novel transcripts.

**Fig. 5:**
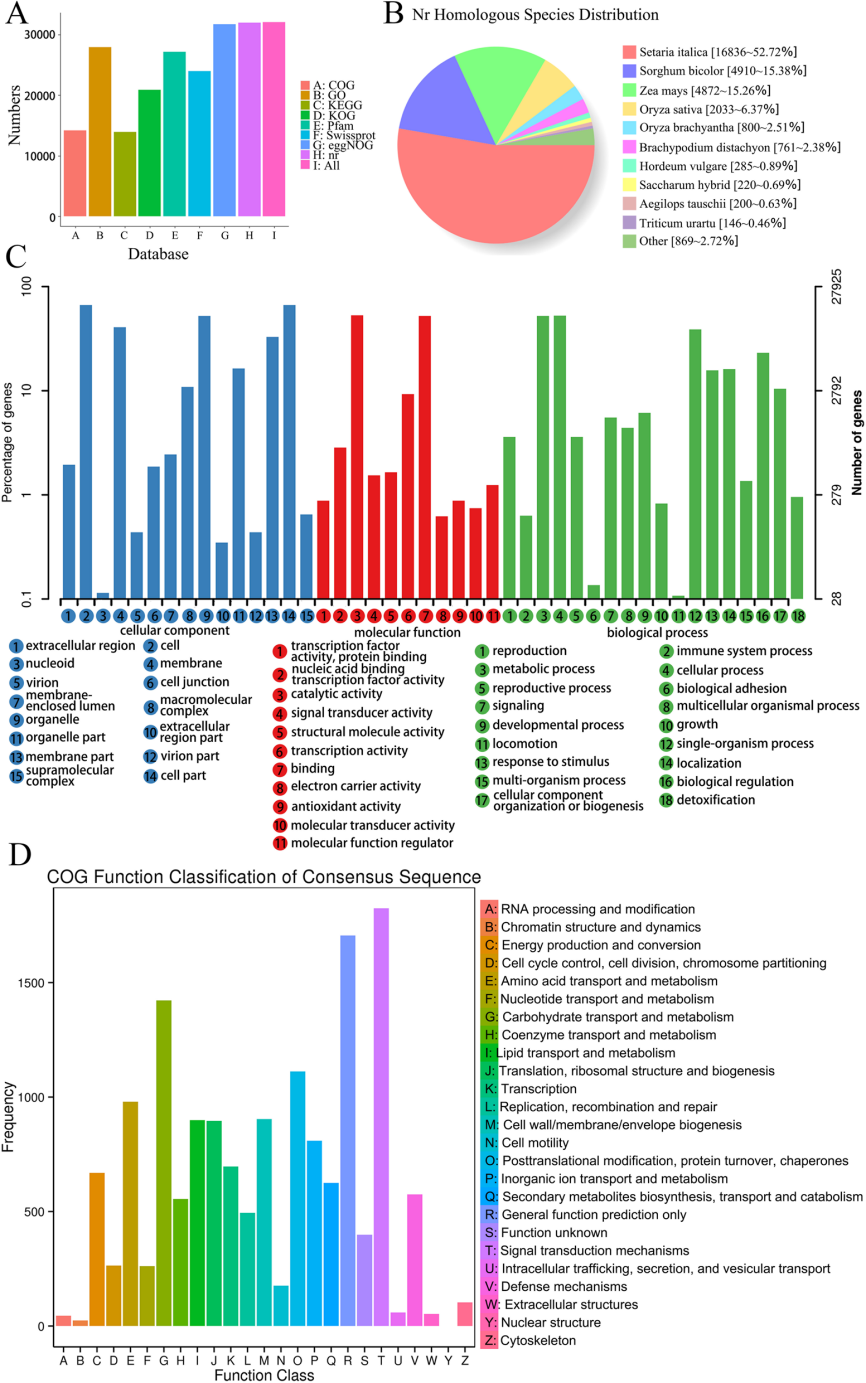
Function annotation of novel transcripts. **A** Function annotation of transcripts in all databases. **B** Nr Homologous species distribution diagram of transcripts. **C** Distribution of GO terms for all annotated transcripts in biological process, cellular component and molecular function. 27,925 transcripts were classified into three groups: “Cellular component,” “Molecular function,” and “Biological processes.” **D** COG function classification of consensus sequences. 24 functional clusters involved in 14,231 novel transcripts were identified

We used BLAST software (version 2.2.26) [23] to compare the 32,948 novel transcript sequences with the NCBI non-redundant protein sequences (NR), Swissprot [24], Kyoto Encyclopedia of Genes and Genomes (KEGG) [25], Gene Ontology (GO) [26], Cluster of Orthologous Group (COG) [27], Eukaryotic Ortholog Groups (KOG) [28], Protein family (Pfam) [29], and eggNOG databases [30], and obtained 32,003 (97.13%) transcript annotations (Fig. 5A, Table S4). We compared 31,935 transcript sequences to the NR database, and the homologous species analysis results showed that the most significant number of transcripts (52.72%) was distributed in *Setaria viridis* (Fig. 5B). GO enrichment analysis showed that 27,925 transcripts were classified into three groups. Genes involved in “Biological processes” were related to metabolic, cellular, single-organism, and biological regulation and response to stimulus processes. Genes involved in the “Molecular function” were mainly associated with binding, catalytic, transporter, transcription factor, and structural molecule activities. Finally, for the “Cellular Component” term, genes were mainly involved in cell, cell part, organelle, membrane, and membrane part (Fig. 5C). To evaluate the completeness of novel isoforms and the validity of the annotations, we identified 24 functional clusters involved in 14,231 novel isoforms using COG analysis (Fig. 5D). The top five functional clusters were “General function prediction only (2676, 18.8%),” “Signal transduction mechanisms (2548, 17.9%),” “Carbohydrate transport and metabolism (2407, 16.91%),” “Posttranslational modification, protein turnover, chaperones (1847, 12.98%),” and “Translation ribosomal structure and biogenesis (1725, 12.12%),” respectively. We used the KEGG database to annotate novel transcripts, and 13,966 transcripts were classified into 136 KEGG pathways. In this study, there were 270 unigenes (8.99%) involved in plant hormones signal transduction pathways that actively accelerated plant senescence.

### Alternative polyadenylation events analysis

**Fig. 6:**
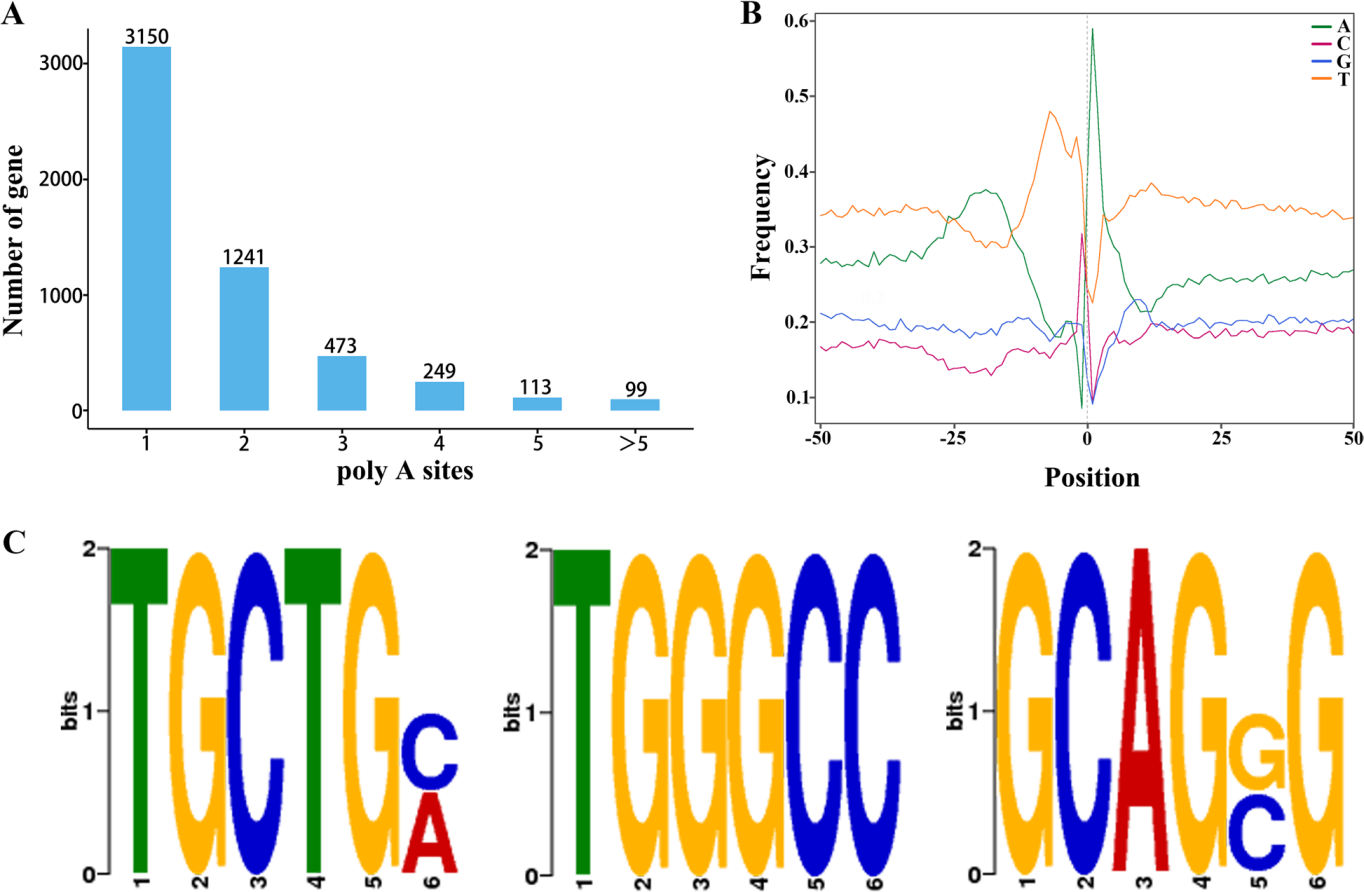
APA analysis. **A** Distribution of the number of poly(A) sites per gene. **B** Nucleotide composition around poly(A) cleavage sites. The relative frequency of a nucleotide is shown as a function of genomic position across all poly(A) cleavage sites detected in our data. **C** MEME analysis of an over-represented motif at 50-nts upstream of the poly(A) site in *Z. japonica* transcripts

The fa. File FLNC sequences were used as the input and, the default parameters were selected to obtain the APA results. We identified 16,102 genes, 5325 of which (containing 66,499 transcripts) had at least one polyadenylation (poly(A) site), and 212 had at least five poly(A) sites (Fig. 6A, Table S5), with an average of 1.75 poly(A) sites. In Table S5, "num sites" correspond to the number of poly(A) sites, and "locations" correspond to the locations of poly(A) sites. Analysis of the nucleotide composition upstream (50 bp) and downstream (50 bp) of the poly(A) cleavage site showed that the upstream of the poly(A) cleavage site in *Z. japonica* was rich in uracil (U), and the downstream was rich in adenine (A) (Fig. 6B); the 50 bp upstream of poly(A) sites of all transcripts was analyzed through MEME for conserved elements. The results showed that there were three conserved motifs (UGCUG, UGGGCC, and GCAGGG, Fig. 6C) upstream of the *Z. japonica* poly(A) cleavage sites.

### Splice isoforms and AS events in Z. *japonica*

**Fig. 7:**
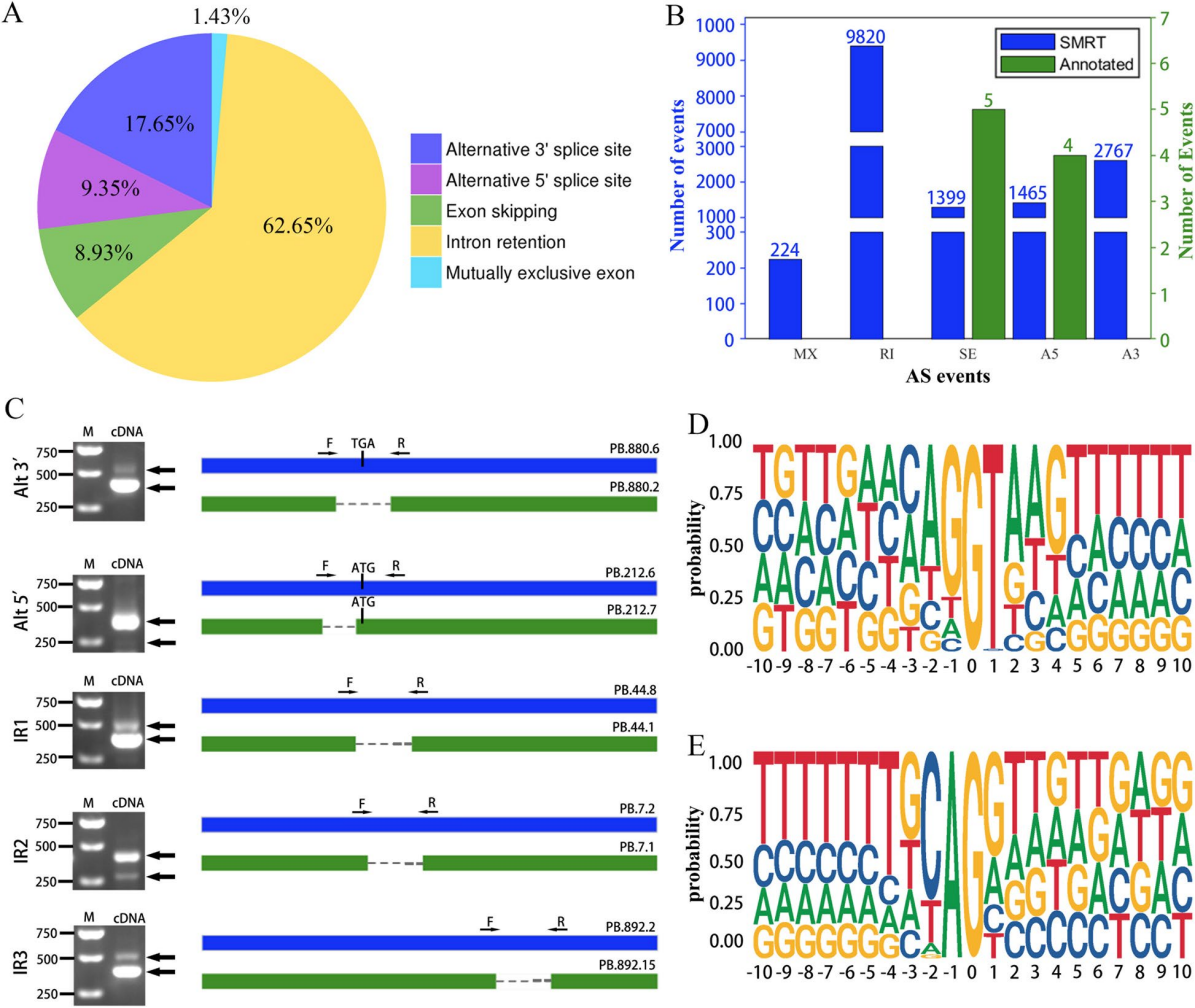
AS events analysis with SMRT sequencing data. **A** Classification of the AS events identified by SMRT sequencing. **B** Comparison of AS events identified between SMRT sequencing data and reference genome. **C** RT-PCR verification of representative AS events. **D** The nucleotide distributions flanking the donor sites. **E** The nucleotide distributions flanking acceptor sites

In our study, 8397 genes consisted of only a single isoform. 7920 to have two or more isoforms. 218 genes consisted of more than ten splice isoforms (Fig. 3D). However, in the reference genome, all 59,271 genes constituted of a single isoform. Therefore, the average number of isoforms per gene (2.27) identified using SMRT sequencing data was significantly higher than that in the reference genome. We detected 15,675 AS events in *Z. japonica* (Table S6). AS events were further classified into five distinct types as follows: 9820 intron retentions, 2767 alternative 3ʹ splice sites, 1465 alternative 5ʹ splice sites, 1399 exon skipping events, 224 mutually exclusive exons. Besides, 62.65% of AS events were intron retentions (Fig. 7A). We randomly selected five genes to validate the accuracy of the splice isoforms detected using SMRT sequencing data (Table S3). We designed primers based on the SMRT sequences for RT-PCR verification. The gel banding pattern (Fig. 7B) and the size of the fragments were consistent with the splice isoforms identified from SMRT data, and DNA sequencing confirmed the accuracy of the SMRT reads. However, only nine AS events were identified in the reference genome, much lower than the number identified through SMRT sequencing data (Fig. 7C).

We also analyzed the consensus donor and acceptor sites of *Z. japonica*, as shown in Fig. 7D-E. AS events identified in our study largely enriched the transcript of the Zoysiagrass genome. There were 18,187 transcripts and 493 genes presented in AS events. To discover key biological processes, we performed GO and KEGG analyses of the transcripts and genes presented in AS events. KEGG analysis showed that “selenocompound metabolism,” “biosynthesis of amino acids,” and “carbon metabolism” were the three most enriched pathways for the genes presented in AS events (Fig. S3). “Pyruvate metabolism,” “carbon metabolism,” “circadian rhythm plant,” “fatty acid degradation,” and “carbon fixation in photosynthetic organisms” were the five most enriched pathways for AS event transcripts (Fig. S4).

### Simple sequence repeats (SSR) analysis

SSR markers of 36,906 transcripts with a length of more than 500 bp were analyzed using MISA, and 16,362 SSRs were identified in 12,081 sequences (Table 3). There were six types of SSRs in the transcriptome, and most of those SSRs were mono-, di-, or tri-nucleotide repeats, and the mono-nucleotide SSRs were the most abundant, accounting for 48.64%. The least abundant type was penta-nucleotide repeats, accounting for 0.15%.

**Table 3:** Prediction of Simple sequence repeats (SSRs) out of transcript datasets

Item	Numbers
Total number of sequences examined	36,906
Total size of examined sequences (bp)	94,358,785
Total number of identified SSRs	16,362
Number of SSR containing sequences	12,081
Number of sequences containing more than 1 SSR	3,251
Number of SSRs present in compound formation	1,058
Mono nucleotide	7,959
Di nucleotide	2,914
Tri nucleotide	5,276
Tetra nucleotide	144
Penta nucleotide	25
Hexa nucleotide	44

### Long non-coding RNA identification

**Fig. 8:**
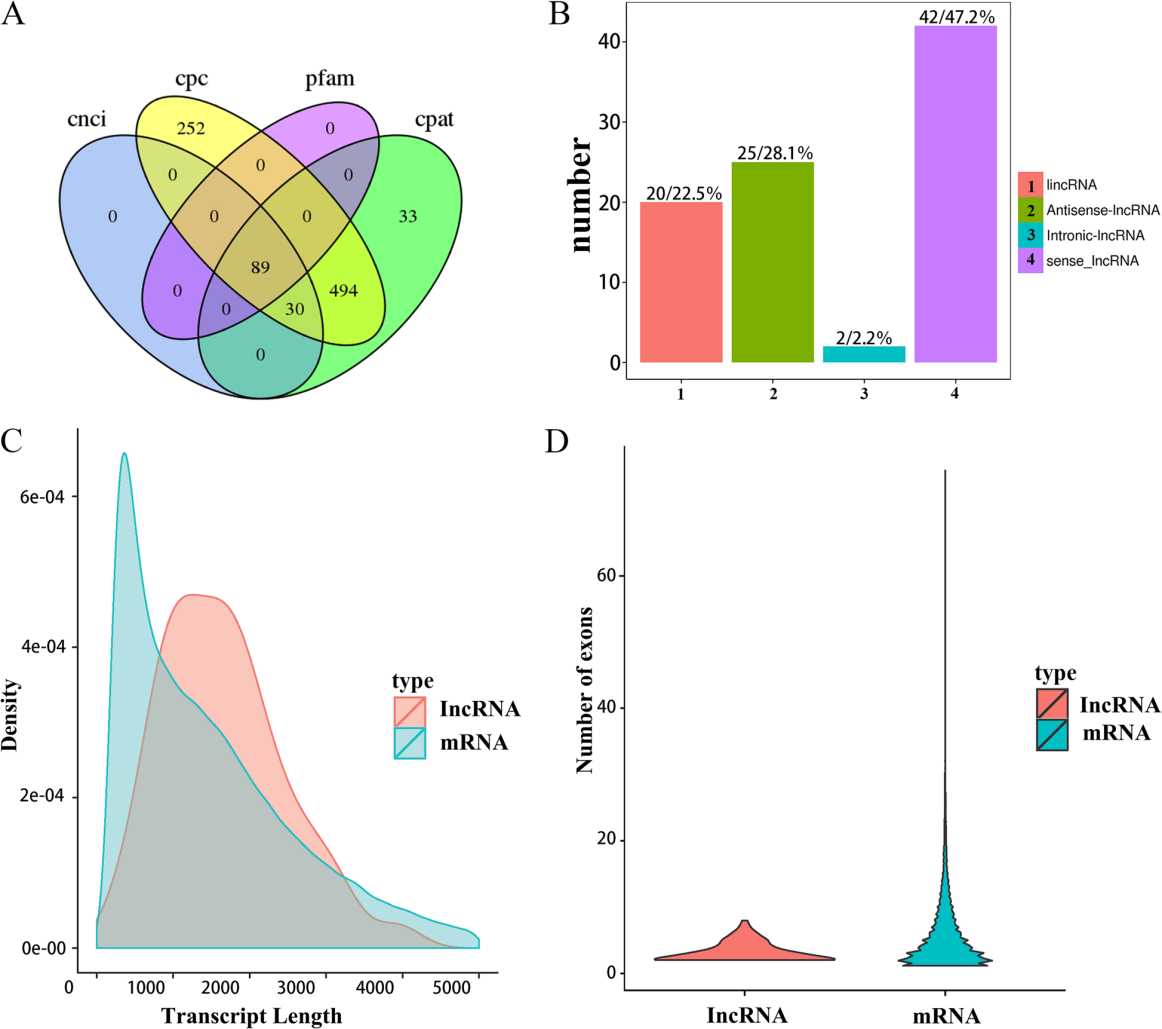
Identification of lncRNAs. **A** Venn diagram of lncRNAs predicted by CNCI, CPC, CPAT and Pfam methods. **B** Classification of the types of the lncRNAs. **C** Density and length distribution of lncRNAs and mRNAs identified in this study. **D** Comparison of exon number of lncRNAs and mRNAs identified in *Z. japonica*

Long non-coding RNAs (lncRNAs) are another important component of the transcriptome. To identify lncRNAs in the PacBio data, we analyzed 32,948 novel transcripts using four methods as follows: CPC, CNCI, CPAT, and Pfam. A total of 89 transcripts were predicted as lncRNAs using the four methods (Fig. 8A). LncRNAs were divided into four types: lincRNAs, antisense lncRNAs, intronic lncRNAs and sense lncRNAs (Fig. 8B). Among them, sense lncRNAs were the most abundant, accounting for 47.2%. Length distribution and exon number analysis of lncRNAs and mRNAs revealed that lncRNAs had longer mean transcript length (1702.91 bp) and fewer exon numbers, whereas the mean length of mRNA was 1593.96 bp (Fig. 8C-D).

### DEG analysis

The Q30 value of Illumina sequencing was more than 94%, indicating that the sequencing data was enough for follow-up analysis. We identified a total of 10,710 DEGs (Fig. S5), and there were 2964 up-regulated and 2759 downregulated genes in groupA (young vs mature). In groupB (mature vs senescent), there were 1851 upregulated and 2187 downregulated genes. The DEGs of groupC (young vs senescent) included 4723 upregulated and 5060 downregulated genes, which were the most abundant.

### Ethylene responses during Z. *japonica* senescence

We randomly selected five upregulated and five downregulated genes from DEGs for quantitative RT-PCR (qRT-PCR) and verified the reliability of transcriptome data by comparing qRT-PCR and next-generation sequencing (NGS) data. R^2^ values are presented to indicate the correlation in Fig. S6. The results showed that the expression of the ten genes involved in plant hormone signaling and photosynthesis in young, mature, and senescent leaves were consistent with the results of the transcriptome analysis. The NGS data was reliable for further investigation.

In this study we analyzed DEGs in young, mature, and senescent leaves (Fig. S5), and the plant hormone signal transduction pathway (270 unigenes) was significantly enriched and correlated positively with aging. We focused on the ethylene pathways including its synthesis and the related signal transduction, and showed the relative transcript levels of genes involved in these pathways using a heatmap (Fig. S7). The results showed that the expression of many genes in the ethylene signal transduction pathway, such as *CTR1*, *SIMKK*, *EIN3*, *EBF1/2*, and *ERF1/2*, changed and most of them were upregulated. In the ethylene synthesis pathway, *SAM*, *ACS*, and *ACC* presented mutations.

### Transcription factor (TF) dynamics during Z. *japonica* senescence

**Fig. 9:**
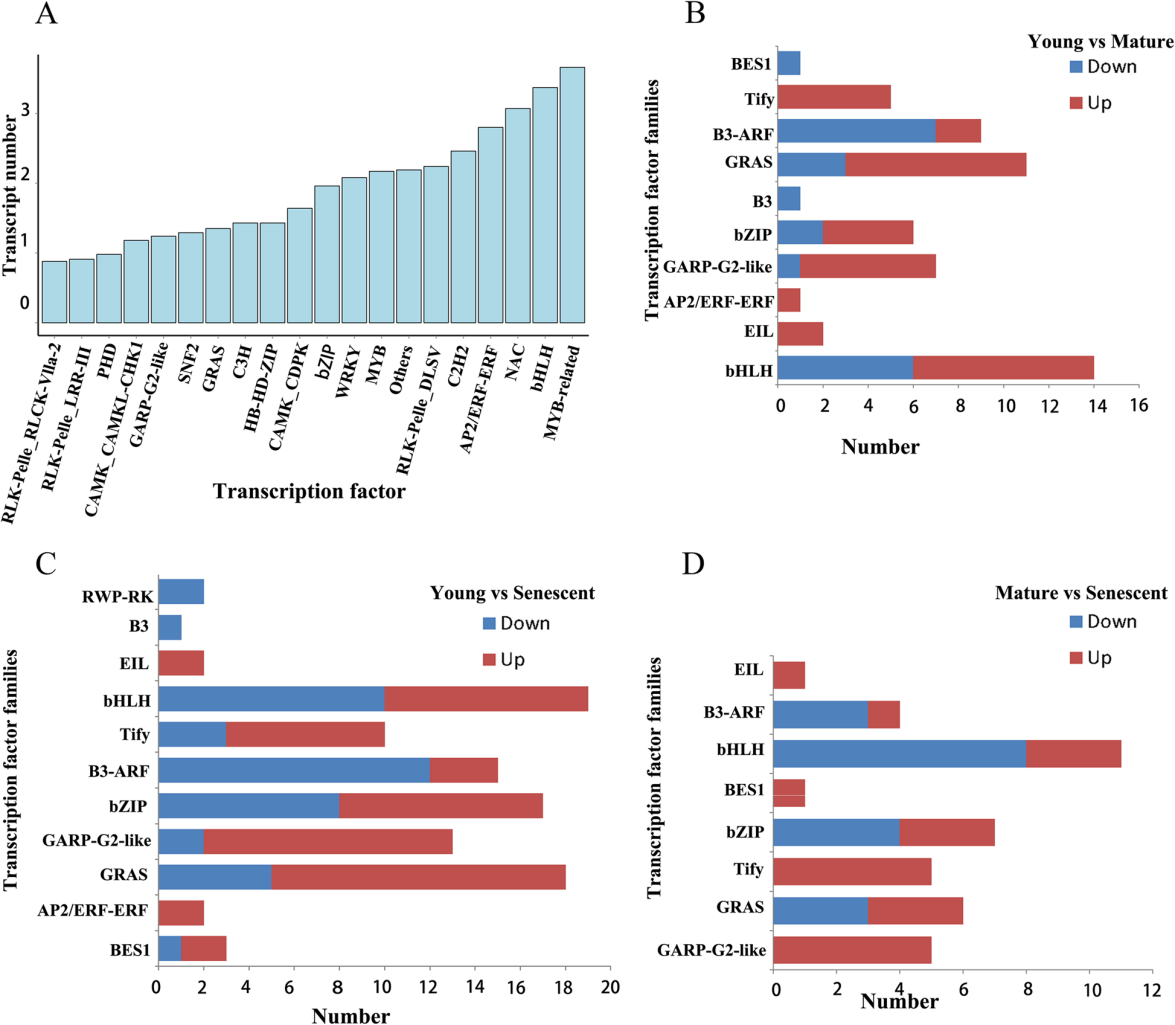
Prediction of transcription factors. **A** Number and family of top 20 transcription factors (TFs) predicted by SMRT sequencing data. **B-D** The differentially expressed TFs Predicted in Young vs Mature, Mature vs Senescent and Young vs Senescent, respectively

TFs play important regulatory roles in plant growth and development. Therefore, the 8035 putative TFs from 209 TF families were predicted using the prediction tool in iTAK. The top five TF families identified were MYB-related (366), bHLH (337), NAC (307), AP2/ERF-ERF (280), C2H2 (246), and the top 20 families identified are shown in Fig. 9A. In addition, we focused on the expression patterns of TFs during the senescence process in *Z. japonica*. We discovered 11 differentially expressed TF families (Fig. 9B-D). The TFs of EIL and AP2/ERF-ERF families were upregulated, and those of B3 and RWP-RK families were downregulated. More upregulated TFs were found in GARP-G2-like, GRAS, and Tify families. More downregulated TFs were found in the B3-ARF TF family.

## Discussion

*Z. japonica* is an important warm-season turfgrass with many excellent traits. Its genome sequence has been published in 2016 [11]. The transcriptome of *Z. japonica* has also been reported by Illumina sequencing in recent years, mainly using NGS technology for gene expression analysis, such as mining the differentially expressed genes between different varieties [13], exploring the tolerance of *Z. japonica* to salt [12, 14], etc. However, the Illumina RNA-seq data frequently cannot accurately obtain or assemble complete transcripts and cannot identify information such as AS, APA, etc. It makes it difficult to understand the deeper meaning of the plant transcriptome. With the advancement of sequencing technology, PacBio SMRT sequencing provides a new way to conduct these studies. The accuracy of the PacBio SMRT CCS model sequencing has been greatly improved, and reliable sequencing data can be obtained without RNA-Seq data correction [31]. The construction of PacBio CCSs and FLNC reads completely avoids the need to assemble short transcriptome reads [31]. Full-length transcriptome information is very useful for plant genome annotation and gene function research.

In this study, we analyzed *Z. japonica* transcripts using the third-generation sequencing technology, according to the latest method for analyzing the PacBio transcriptome data [17–19, 32]. A total of 56,231 consensus isoforms were generated. 37,056 high-quality non-redundant transcripts were obtained through mapping against the reference genome. The high capacity of PacBio transcriptome sequencing to generate full-length transcript sequences is probably result from the long sequencing length. Previous transcriptome studies have also pointed out that PacBio SMRT sequencing can identify full-length and relatively long transcript sequences [1, 17–19]. In this study, the number of transcripts (> 3000 bp) identified with SMRT sequencing data was significantly higher than that provided by the transcript annotations of the reference genome.

The AS of pre-mRNAs constitutes the diversity of the eukaryotic transcriptome, which determines the coding capacity of the genome and the mechanism of gene regulation [33]. Previous reports have shown that fusion transcripts are associated with AS [34]. SMRT sequencing enabled the identification of the structure of fusion transcripts and the complexity of AS events [34, 35]. Only nine AS events were identified in the *Z. japonica* genome, but we identified 15,675 AS events and 254 fusion transcripts through SMRT sequencing, which enriched the transcript information of the *Z. japonica* genome. In addition, the majority of the AS events in *Z. japonica* were intron retentions, which is consistent with reports on *Arabidopsis*, sorghum, and maize [33, 34, 36].

lncRNAs play an important regulatory role in a variety of biological processes [37, 38]. Our study predicted 89 lncRNAs with an average length of 1702.91 bp using SMRT sequencing, which is longer than those of *Zea mays* (463 bp), *Miscanthus lutarioriparius* (683 bp), and *Trifolium pratense* (665.39 bp) [20, 34, 39]. It is worth mentioning that the average transcript length of lncRNAs is longer than that of mRNAs (1593.96 bp), which is inconsistent with previous reports on other plants [21, 38, 39].

The full-length transcriptome can be used to effectively analyze the exon–intron structure [1]. By comparing the exon–intron structure of transcripts, we found that SMRT sequencing had obvious advantages in mining transcripts containing more than seven exons. The same advantage is also reflected in the analysis of intron structure of SMRT sequencing data. The aforementioned results enrich the transcript information of the *Z. japonica* genome. The genes annotated in the *Z. japonica* genome were all of a single isoform. While SMRT sequencing data provided information on 7920 genes containing two or more splicing isoforms, 218 genes containing more than ten isoforms, which effectively supplemented the genomic information.

Compared with the thousands of cDNAs previously reported, approximately 67.4% of the FLNC reads generated through this sequencing carried the complete ORF. In this study, 913 novel gene loci and 1.274 novel genes were identified. More than 99.1% of the newly annotated genes were compared to the homologs in the database (Table S7). This effectively improved genomic annotation. We found 32,948 novel transcripts and 97.1% of them were successfully annotated, which did not only enrich the transcription information based on ethnic sequences but also facility further functional studies of key genes.

Studies have shown that by analyzing the most representative differential gene data, most of the information in the entire transcriptome can be captured [40]. Illumina sequencing of leaves at three different developmental stages contributes to study the molecular mechanism of *Z. japonica* senescence. Senescence represents the last developmental stage of leaves, decreasing the commercial value of turf and forage grasses [41]. Currently, there are rare studies on the aging of *Z. japonica* using SMRT and NGS analyses. Previous studies have mostly focused on the differential expression and response mechanisms of genes under abiotic stress and the functions of individual genes [9, 12, 14]. The physiological and molecular mechanisms of Zoysiagrass senescence remain largely unknown.

In previous reports, bHLH, GRAS, bZIP, B3-ARF, and GARP-G2-like family of TFs have been widely reported as key factors in plant responses to biotic and abiotic stresses [42–46]. These TFs showed different expression patterns in the leaves of *Z. japonica* at different senescence stages, indicating that they play different roles in *Z. japonica* senescence. Further studies are warranted to explore the function and regulation of transcription factors through transgenic approaches. This study used SMRT and NGS technology, combined with physiological data, to explore the physiological and molecular mechanisms of *Z. japonica* senescence. 270 DEGs were significantly enriched in plant signal transduction pathways. Ethylene synthesis and related signal transduction pathways interact to form a complex network that plays an important role in plant senescence. The senescence of *Z. japonica* is mainly accompanied by the following metabolic processes: 1) Inhibition of photosynthesis and reduction in photosynthetic capacity; 2) regulation of plant hormones and signal transduction; 3) activation of the antioxidant defense enzymes defense; 4) osmotic stress. The proposed regulatory machinery of *Z. japonica* senescence is presented in Fig. S8. To the best of our knowledge, this is the first study to use updated transcriptome data to conduct a transcriptome-wide study on *Z. japonica* senescence.

## Conclusion

We provide the full-length transcriptome of *Z. japonica* using the PacBio SMRT sequencing method. We identified 56,228 high-quality transcripts, and predicted 15,675 AS events, 8035 transcription factors, 5325 APA sites and 89 lncRNAs. Furthermore, NGS data analysis showed the molecular mechanisms of *Z. japonica* senescence. These discoveries have expanded the knowledge on the *Z. japonica* genome, which will enable further research at the transcriptome level and provide a theoretical basis for the selection and breeding of new *Z. japonica* varieties.

## Methods

### Plant material and growth conditions

*Z. japonica* seeds (cv. Zenith) purchased from Patten Seed Company (Lakeland, GA, USA) were sown in Klasmann TS1 peat substrate (Klasmann-Deilmann GmbH, Geeste, Germany) and then plants were cultivated at 28/25 °C (day/night) with a 14 h photoperiod and average photosynthetic active radiation of 400 µmol m^−2^ s^−1^ in climate chambers. The plants were watered once a week with Hoagland nutrition solution. Young, mature, and senescent *Z. japonica* leaves were harvested in September 2020 as described in our previous study [10]. In addition, roots, stolons and flowers were collected. All samples were quickly frozen in liquid nitrogen and stored at -80 °C for further experiments.

### Physiological determinations

Leaves of *Z. japonica* from three different developmental stages (young, mature, and senescent) were used for the physiological determinations. Chlorophyll content was determined according to a previously reported protocol [9]. Electrolyte leakage (EL) was examined using a previously described method [47]. We determined the fresh, turgid, and dry weights of leaves to calculate the relative leaf water content. The plant soluble sugar assay (KT-2-Y), ascorbate peroxidase assay (APX-2-W), catalase assay (CAT-2-W), and peroxidase assay (POD-2-Y) kits were purchased from Suzhou Comin Biotechnology Co., Ltd., Suzhou, China, to determine the content of soluble sugars and antioxidant enzyme activities, including ascorbate peroxidase, catalase, and peroxidase.

Four indexes were used to evaluate photochemical efficiency, including the Pn, Ci, Gs and Tr, using a photosynthetic system (Li6400XT, Li-Cor, USA). ELISA (H251) and ELISA (H602-1), kits were obtained from Nanjing Jiancheng Bioengineering Institute, Nanjing, China, and were used to examine ABA and IAA contents, respectively. All experiments in this study included at least three biological replicates. PCA was used to classify nine samples based on the physiological results.

### Full-length library construction and PacBio SMRT sequencing

For SMRT sequencing, we mixed RNA from roots, stolons, flowers and leaves at three different developmental stages into one sample and constructed a sequencing library without size selection. The SMARTer™ PCR cDNA Synthesis kit (TaKaRa, Dalian, China) was used to synthesize full-length cDNA, and cDNA was not size selected. After library quality was confirmed, we used the PB PacBio Sequel II platform to perform full-length transcriptome sequencing. The analysis to obtain the full-length transcriptome mainly consisted of three stages [48]. First, CCS sequences were extracted and polished from raw reads with a minimum full pass of 3 and minimum predicted accuracy of 0.9. Next, FLNC transcripts were determined by searching for the CCS sequence poly(A) tail signal using 5’ and 3’ cDNA primers. Finally, FLNC sequences were clustered to obtain consensus isoforms, high quality (HQ, transcripts with greater than 99% accuracy), and low quality (LQ) transcripts.

### Illumina cDNA library construction and sequencing

For Illumina sequencing, we constructed nine cDNA libraries (three biological replicates for leaves at three different developmental stages). After the libraries passed quality control, they were sequenced on the Illumina NovaSeq6000 platform (San Diego, CA, USA). The specific quality control of sequencing data was as follows: Cut the sequencing adapter and primer sequence in Reads and filter LQ value data to ensure data quality. This step was performed to obtain HQ reads (clean data). At the same time, the Q30, GC content, and sequence duplication level of clean data were calculated. Next, HISAT2 [49] was used to align clean reads with a reference genome to obtain positional information on the reference genome or gene.

### Genome mapping and AS events prediction

Consensus sequences, FLNC sequences, and non-redundant transcripts were mapped to the *Z. japonica* reference transcript sequences (http://zoysia.kazusa.or.jp) using GMAP [50]. Mapped reads were further collapsed using cDNA Cupcake software (https://github.com/Magdoll/cDNA_Cupcake/wiki) with minimum coverage of 0.85 and minimum identity = 0.9. A 5’ difference was not considered when redundant transcripts were collapsed. AS event recognition and comparison were performed using the ASTALAVISTA software [51]. Data were compared between SMRT sequencing and the transcript annotations of reference genome, including transcript length and number distribution, the distribution of the number of transcripts generated by a single gene, the distribution of the number of exons in the transcript, and the distribution of the number of introns in the novel gene.

### Fusion transcripts, alternative polyadenylation sites, and simple sequence repeats analysis

The 50 bp upstream of the transcript's poly(A) site was analyzed with MEME [52]. In addition, the transcript genome was analyzed across regions to identify the fusion transcript. The fusion transcripts that included two or more loci were identified, minimum coverage for each locus was 5%, minimum coverage was 1 bp, minimum coverage was 0.95, and the distance between two loci was at least 10 kb. Gene structure and APA analyses were conducted using TAPIS pipeline software [33]. In addition, we used the MISA software to identify the SSR of the transcriptome [53].

### Identification of TFs and lncRNAs

The TFs were predicted using the iTAK software [54] with putative protein sequences. Four computational approaches, CPC [55], CNCI [56], CPAT [57], and Pfam [29], were combined to sort non-protein-coding RNA candidates from putative protein-coding RNAs in the transcripts. First, putative protein-coding RNAs were filtered using the minimum length and exon number threshold. Then, transcripts longer than 200 bp and containing more than two exons were selected as lncRNA candidates and further screened using CPC/CNCI/CPAT/Pfam, which distinguished the protein-coding from the non-coding genes.

### Prediction of the coding region of novel genes

To predict ORFs and find potential coding sequences, as well as the corresponding amino acid sequence of the novel transcripts, obtained in the AS analysis, TransDecoder (v3.0.0) software [58] was used. We predicted the putative protein sequences and statistically analyzed the length distribution of full-length transcripts (containing complete ORFs as well as 5ʹ- and 3ʹ-UTR).

### Gene functional annotation, GO, and KEGG enrichment analysis

To investigate the functions of all novel transcripts, gene function was annotated based on the following databases: NR, GO, KEGG, KOG/COG/eggNOG, Swiss-Prot (a manually annotated and reviewed protein sequence database), and Pfam. In addition, GO enrichment analysis of DEGs was implemented with using GOseq R packages based on Wallenius non-central hypergeometric distribution [59], which adjusts for gene length bias in DEGs. We used KOBAS software (version 2.0) [60] to test the statistical enrichment of DEGs in KEGG pathways. In addition, we performed GO and KEGG enrichment analysis on the genes and transcripts that presented AS events to identify biological processes.

### RT-PCR validation of novel genes, alternative splicing events, and fusion transcripts

For PCR validation of novel genes, fusion transcripts, and AS events, primers were designed using DNA-MAN (version 6.0) and Primer Premier (version 6.0). All primers used in the RT-PCR analysis are shown in Table S3. RT-PCR analysis was performed using cDNA as a template in a Bio-Rad C1000 Touch™ Thermal Cycler. The PCR products were monitored using 1% agarose gel electrophoresis, and the electrophoresis results were photographed.

### Quantification of gene expression levels and analysis of differential gene expression

We used the fragments per kilobase of transcript per million fragments mapped (FPKM) method to calculate the expression level of transcripts. After detecting correlations between bio-replicates using the Pearson Correlation Coefficient, we used the DESeq software to analyze differential expression [61]. The DEGs were detected using the criteria of fold change of ≥ 2 and FDR of < 0.01. The p-value was corrected via the Benjamini–Hochberg correction method. Finally, we obtained the DEGs. We compared the differences in gene expression levels in the two groups of samples and the statistical significance of the differences using volcano plots.

### qRT-PCR verification of DEGs

Reverse transcription was performed using PrimeScript™ Reverse Transcriptase (TaKaRa, Dalian, China) and the product was purified using the Cycle Pure kit (OMEGA, Georgia, USA). Five upregulated and five downregulated DEGs were randomly selected. qRT-PCR primers were designed using Primer Premier 6, and their specificity was verified through PCR (Table S8). qRT-PCR analysis was conducted on a Bio-Rad CFX Connect™ Real-Time System using SYBR® Premix Ex Taq™ II (TaKaRa, Dalian, China). *Z. japonica β-actin* (GenBank accession No. GU290546) was selected as the housekeeping gene. All gene expression analyses were performed using three biological replicates. We calculated the relative expression level of genes using the 2^−ΔΔCT^ method [10].

## Supplementary Information

**Additional file 1: Figure S1. **PCA analysis of nine samples.

**Additional file 2: Figure S2. **Summary of SMRT sequencing. (A) CCS read length distribution. (B) FLNC sequences read lengthdistribution. (C) Consensus isoforms read length distribution.

**Additional file 3: Figure S3. **KEGG enrichment of in the genespossessed AS events.

**Additional file 4: Figure S4. **KEGG enrichment of in the transcriptspossessed AS events.

**Additionalfile 5: Figure S5.** DEG analysis based on the Illumina sequencingdata. A–C Volcano plot and KEGG enrichment of the DEGs identified in young vsmature, mature vs senescent, and young vs senescent, respectively.

**Additional file 6: Figure S6. **qRT-PCRresults of the expression levels of 10 randomly selected different genes inyoung, mature and senescent leaves. R^2^ represents the correlationbetween qRT-PCRand NGS results.

**Additional file 7: Figure S7. **DEGsinvolved in and ethylenebiosynthetic and signal transduction pathways. A. Ethylene signal transductionpathway. B. Ethylene synthesis pathway. In the pathway, red represents up-regulatedgenes, green represents down-regulated genes, and blue represents bothup-regulated and down-regulated genes.

**Additional file 8: Figure S8. **Aproposed regulating machinery model ofsenescence in *Z. japonica*.

**Additional file 9: Table S1. **Physiological change of *Z. japonica* inresponses to senescence.

**Additional file 10: Table S2. **Summaryof fusion transcripts in *Z. japonica*.

**Additional file 11: Table S3. **Primers used for RT-PCR validation.

**Additional file 12: Table S4. **Listof novel transcripts and their annotation.

**Additional file 13: Table S5. **Summaryof APA sites.

**Additional file 14: Table S6. **Summary of AS events.

**Additional file 15: Table S7. **Listof novel genes and their annotation.

**Additional file 16: Table S8. **Primersused for qRT-PCR validation.

**Additional file 17: Figure S9. **Supplementary Original Full length of Figure 4.

**Additional file 18. Figure S10 **Supplementary original of Figure 7C.

## Data Availability

The PacBio SMRT reads and the Illumina NGS reads generated in this study were submitted to the BioProject database of the National Center for Biotechnology Information (accession numbers PRJNA774118, https://dataview.ncbi.nlm.nih.gov/object/PRJNA774118?reviewer=eu0drl4chtf3fp373avtd9rs3t; PRJNA775807, https://dataview.ncbi.nlm.nih.gov/object/PRJNA775807?reviewer=r2nmi84k3knoo4ngtsqnt2nuhb).

## Abbreviations

APA: Alternative Polyadenylation Sites
AS: Alternative Splice Events
CCS: Circular Consensus Sequences
CDS: Coding Region Sequences
COG: Clusters of Orthologous Groups of Proteins
DEG: Differentially Expressed Gene
FLNC: Full-Length Non-Chimeric Reads
FPKM: Fragments Per Kilobase of Transcript Per Million Fragments Mapped
GO: Gene Ontology database
KEGG: Kyoto Encyclopedia of Genes and Genomes
KOG: Eukaryotic Ortholog Groups
lncRNA: Long Non-coding RNA
NR: NCBI non-Redundant Protein Sequences
NGS: Next-Generation High Throughput Sequencing
Nr: Non-redundant protein database
ORF: Open Reading Frame
PCA: Principal Component Analysis
Pfam: Protein Family
RT-PCR: Reverse Transcription Polymerase Chain Reaction
SMRT: Single-Molecule Real-time
SSR: Simple Sequence Repeats
TF: Transcript Factor
