# Supplementary figures and images for: Single-molecule long-read sequencing analysis improves genome annotation and sheds new light on the transcripts and splice isoforms of Zoysia japonica
Source: BMC Plant Biol. 2022 May 26;22:263. doi: 10.1186/s12870-022-03640-7 (PMC9134579; doi:10.1186/s12870-022-03640-7)

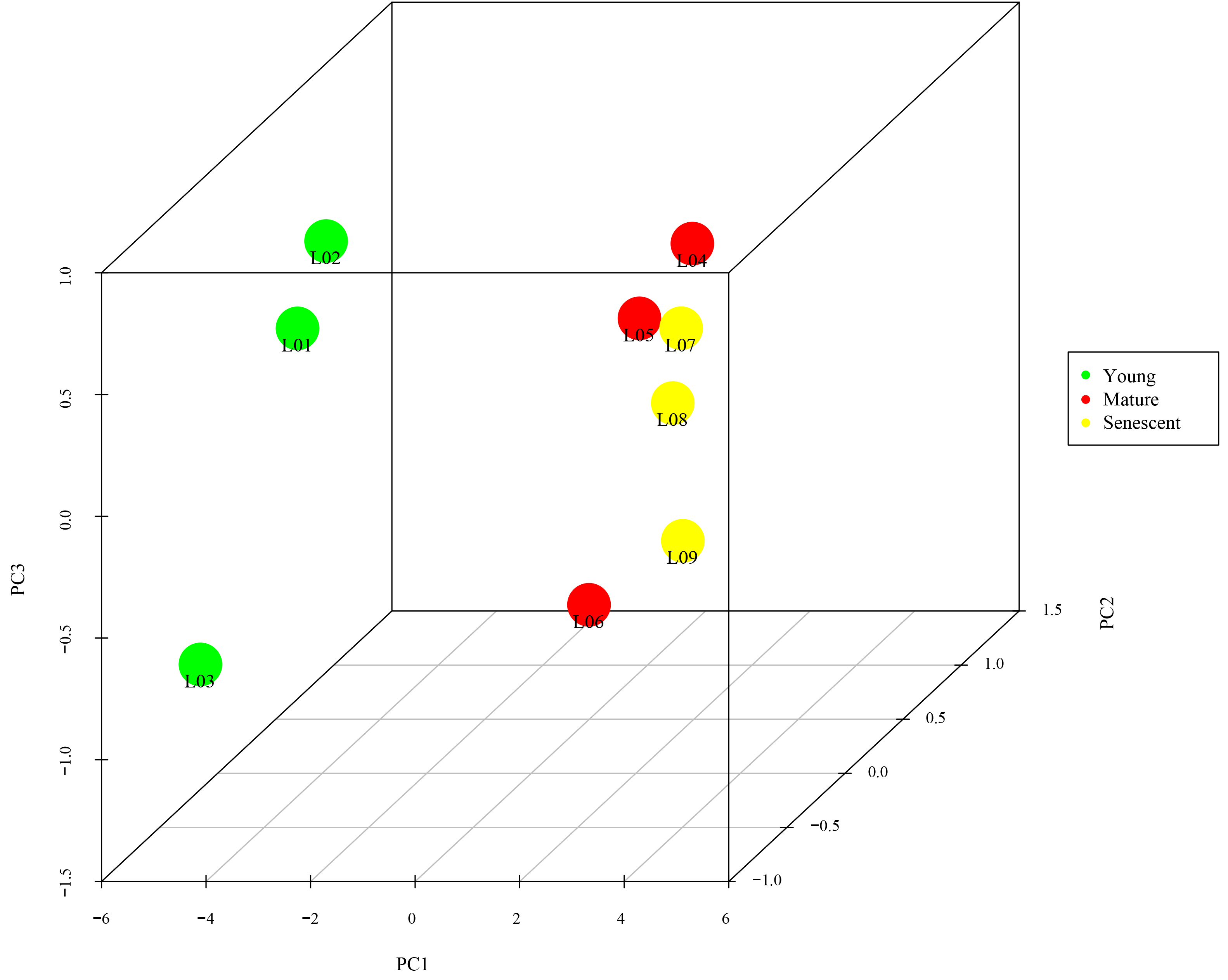

Supplement: Supplementary file 1 — Additional file 1: Figure S1. PCA analysis of nine samples. [file 12870_2022_3640_MOESM1_ESM.png]

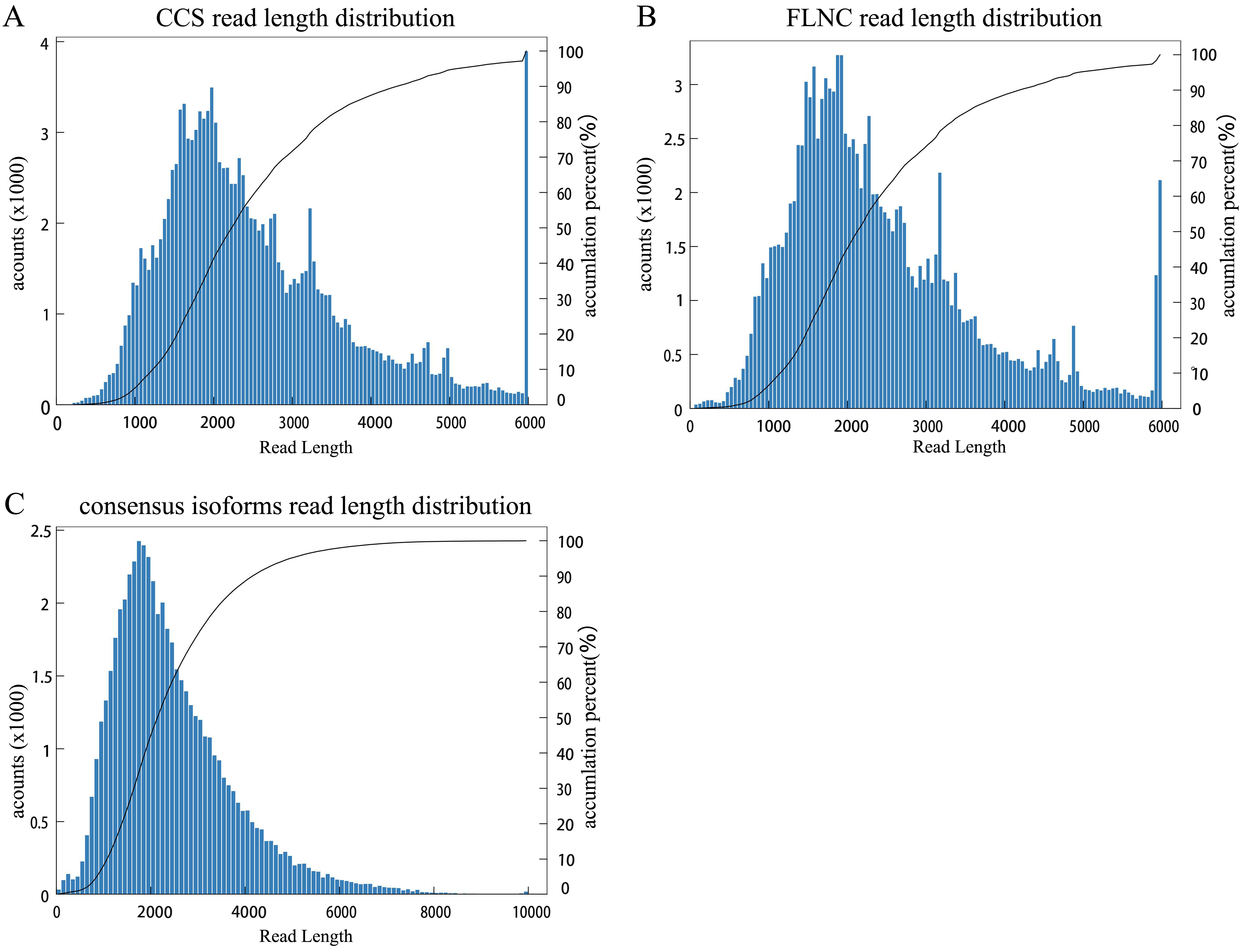

Supplement: Supplementary file 2 — Additional file 2: Figure S2. Summary of SMRT sequencing. (A) CCS read length distribution. (B) FLNC sequences read lengthdistribution. (C) Consensus isoforms read length distribution. [file 12870_2022_3640_MOESM2_ESM.png]

# Statistics of Pathway Enrichment

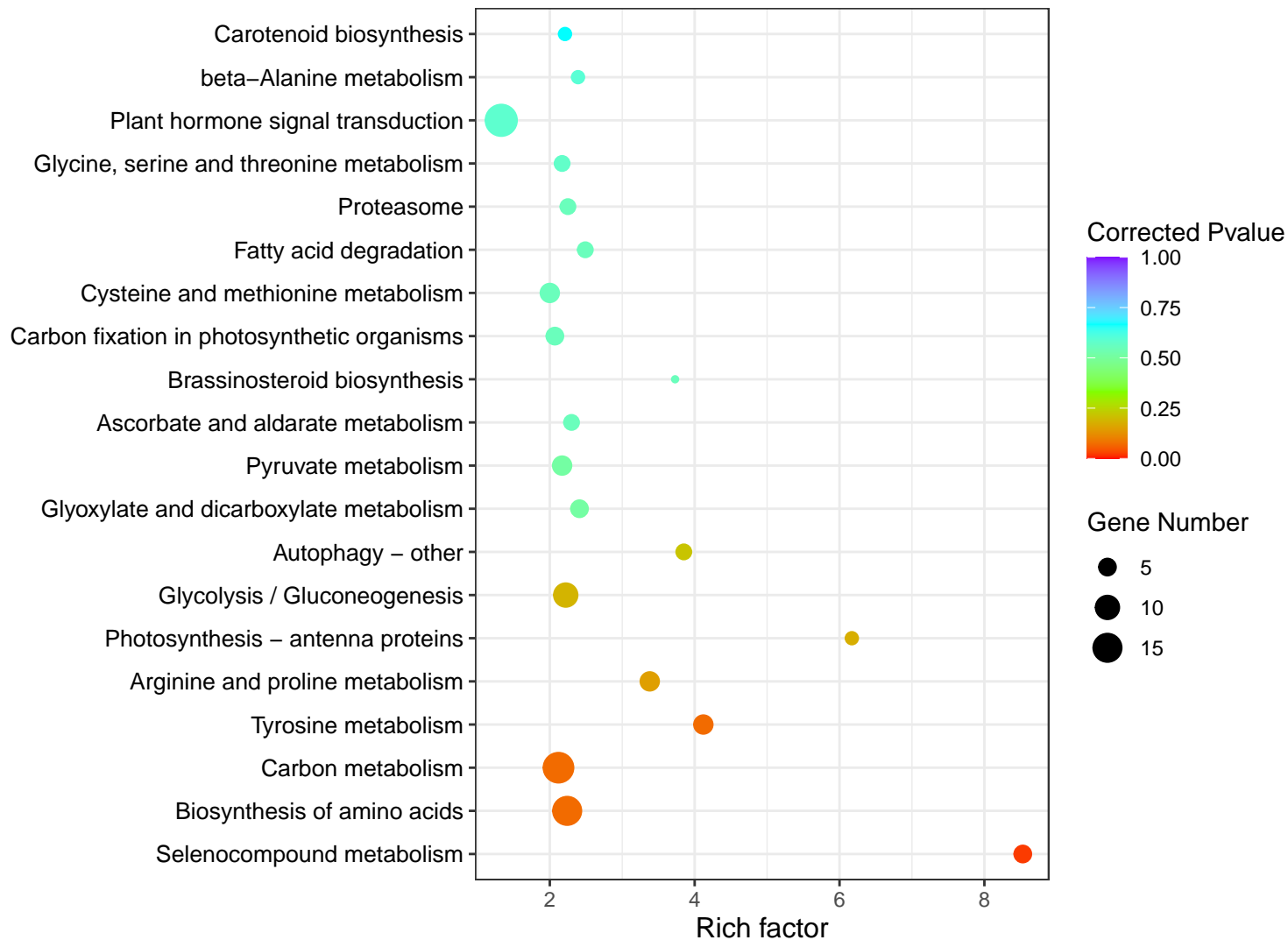

Supplement: Supplementary file 3 — Additional file 3: Figure S3. KEGG enrichment of in the genespossessed AS events. [file 12870_2022_3640_MOESM3_ESM.pdf]

# Statistics of Pathway Enrichment

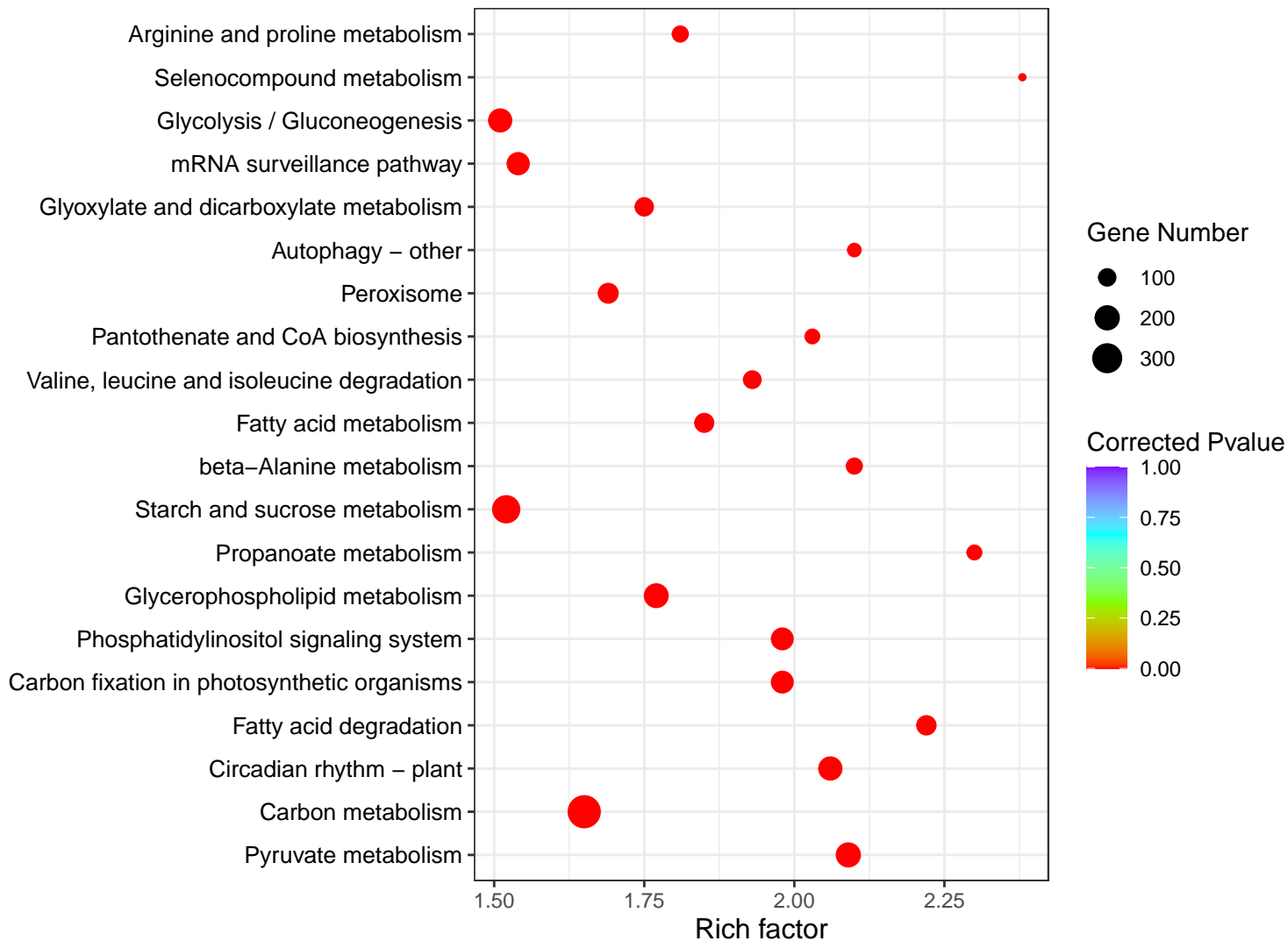

Supplement: Supplementary file 4 — Additional file 4: Figure S4. KEGG enrichment of in the transcriptspossessed AS events. [file 12870_2022_3640_MOESM4_ESM.pdf]

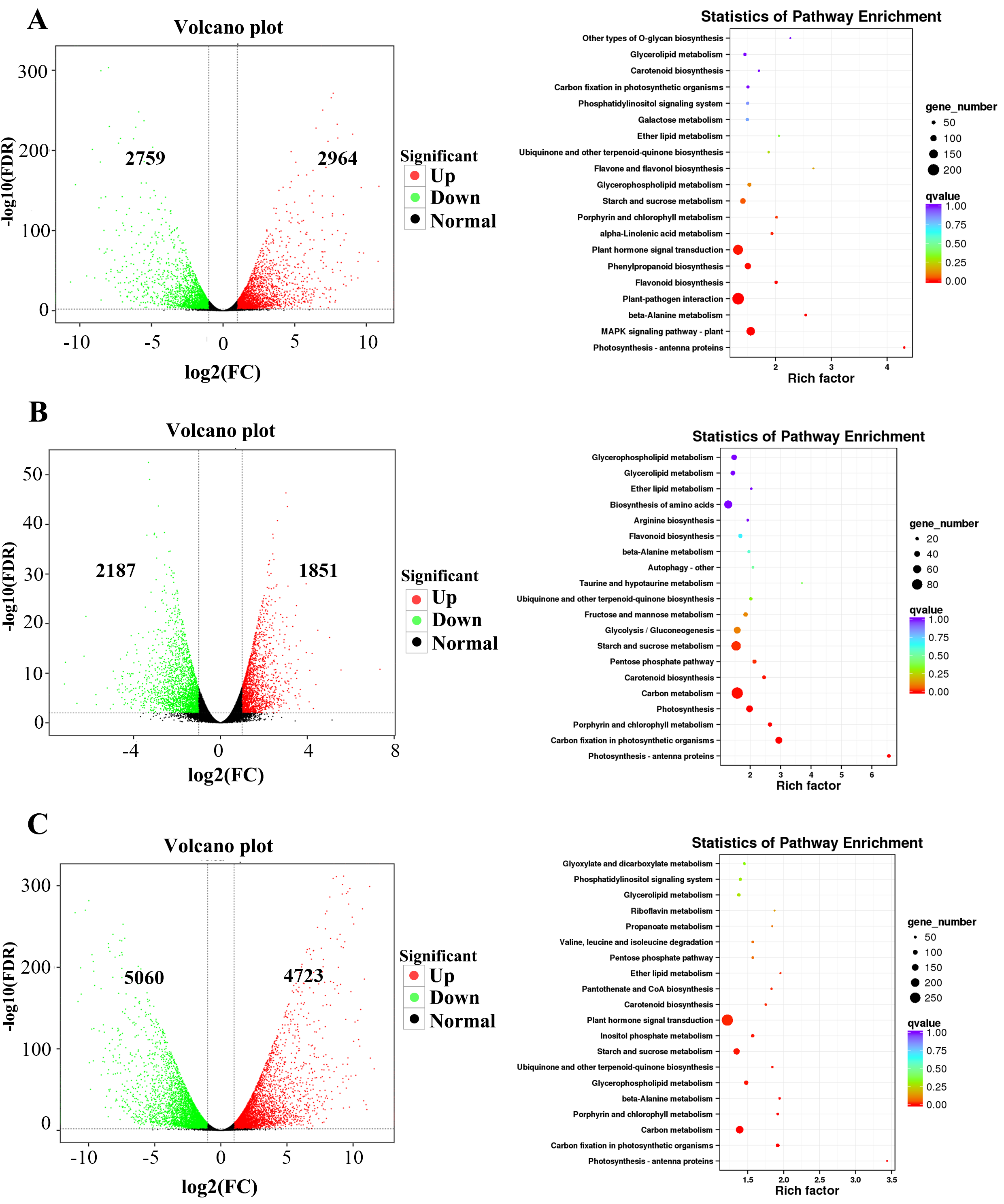

Supplement: Supplementary file 5 — Additionalfile 5: Figure S5. DEG analysis based on the Illumina sequencingdata. A–C Volcano plot and KEGG enrichment of the DEGs identified in young vsmature, mature vs senescent, and young vs senescent, respectively. [file 12870_2022_3640_MOESM5_ESM.png]

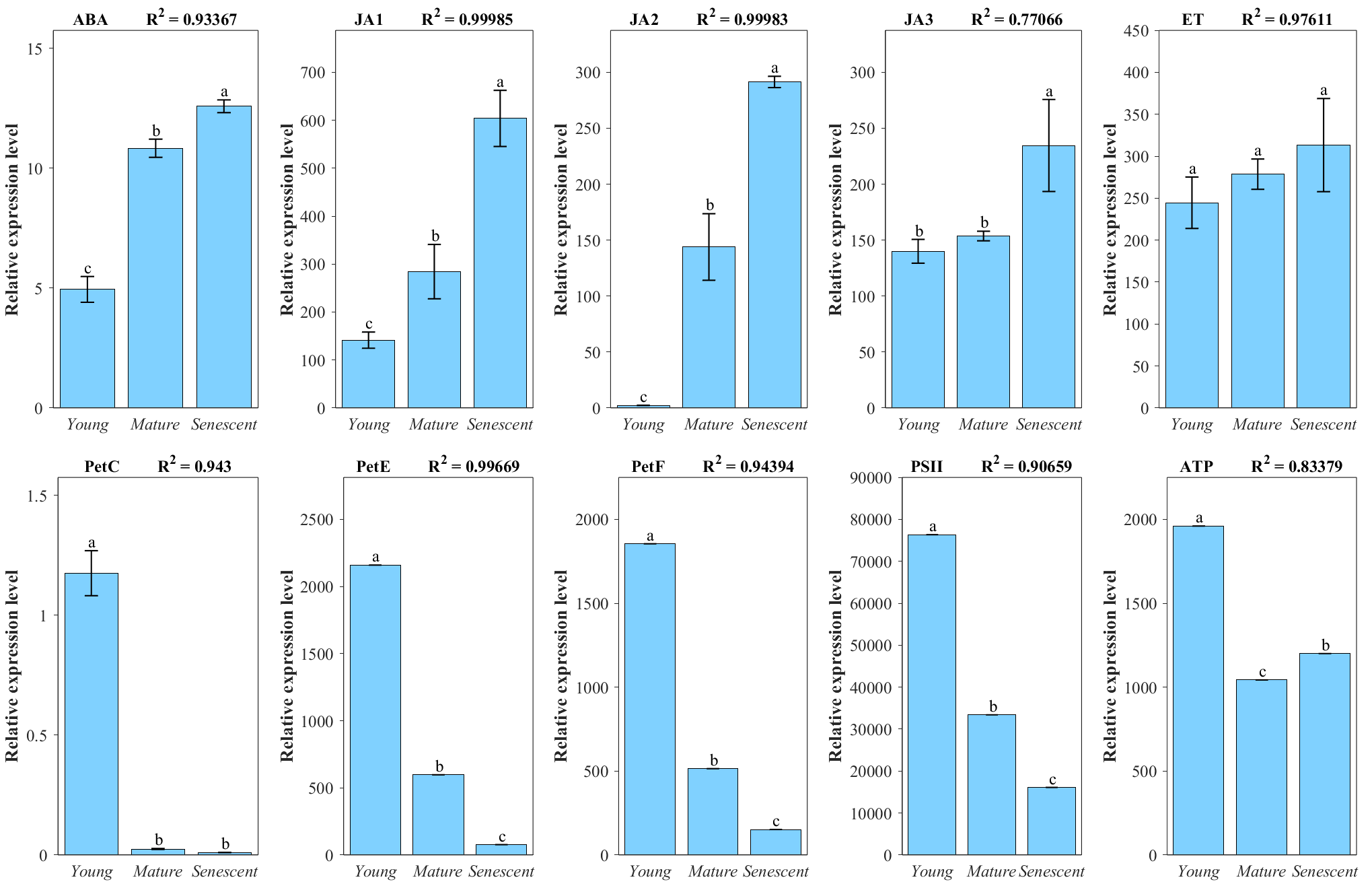

Supplement: Supplementary file 6 — Additional file 6: Figure S6. qRT-PCRresults of the expression levels of 10 randomly selected different genes inyoung, mature and senescent leaves. R2 represents the correlationbetween qRT-PCRand NGS results. [file 12870_2022_3640_MOESM6_ESM.png]

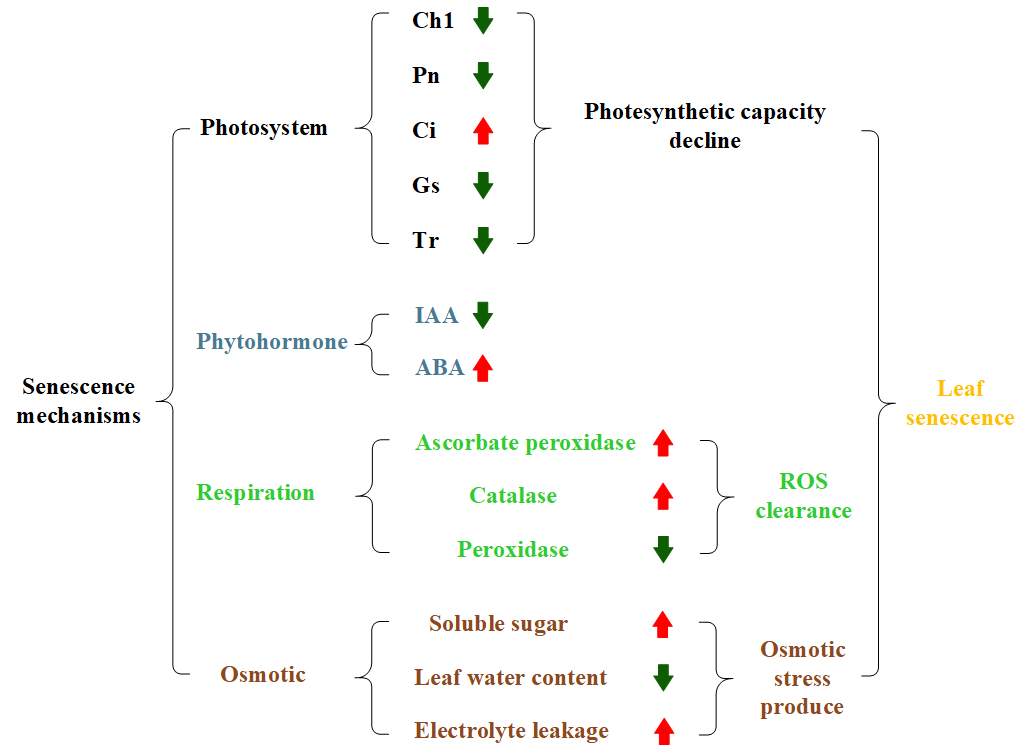

Supplement: Supplementary file 8 — Additional file 8: Figure S8. Aproposed regulating machinery model ofsenescence in Z. japonica. [file 12870_2022_3640_MOESM8_ESM.png]

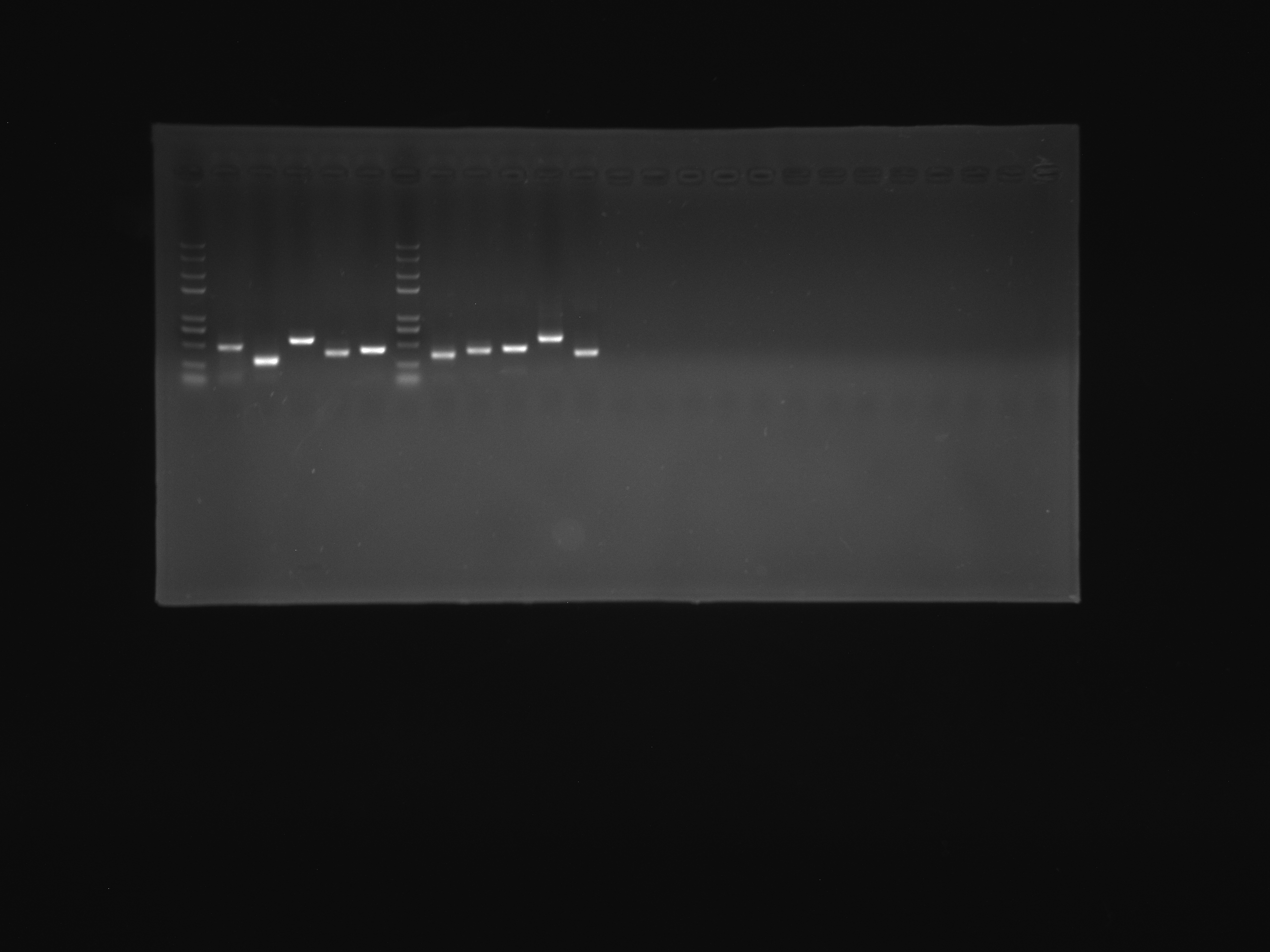

Supplement: Supplementary file 17 — Additional file 17: Figure S9. Supplementary Original Full length of Figure 4. [file 12870_2022_3640_MOESM17_ESM.tiff]

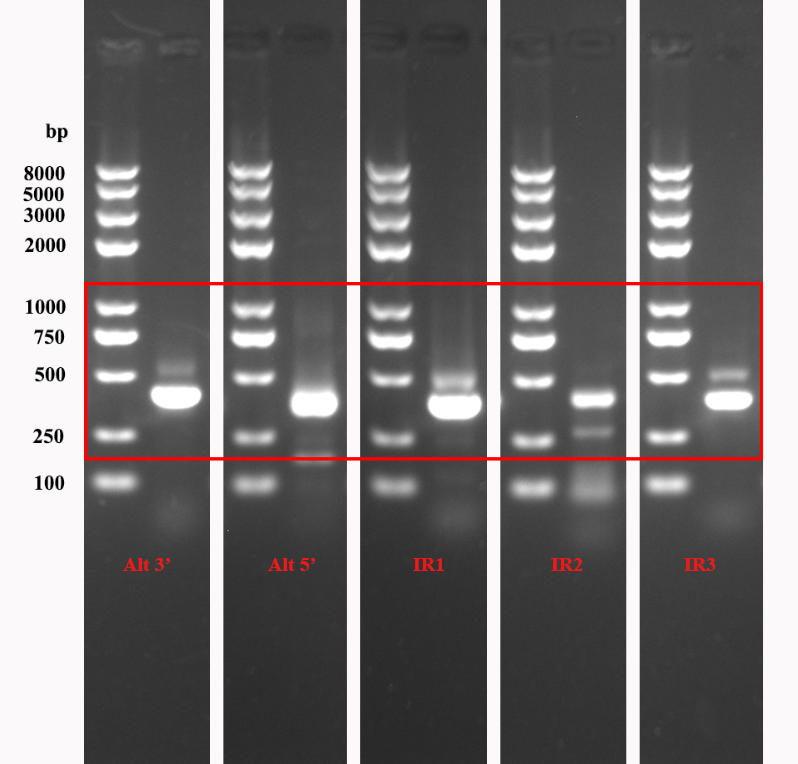

Supplement: Supplementary file 18 — Additional file 18. Figure S10 Supplementary original of Figure 7C. [file 12870_2022_3640_MOESM18_ESM.tif]
