# Supplementary material for: Single-molecule long-read sequencing analysis improves genome annotation and sheds new light on the transcripts and splice isoforms of Zoysia japonica
Source: BMC Plant Biol. 2022 May 26;22:263. doi: 10.1186/s12870-022-03640-7 (PMC9134579; doi:10.1186/s12870-022-03640-7)
Supplement: Supplementary file 9 — Additional file 9: Table S1. Physiological change of Z. japonica inresponses to senescence. [file 12870_2022_3640_MOESM9_ESM.docx]

|  | Chl | Pn | Gs | Tr | Ci | IAA | ABA | APX | CAT | POD | RWC | sugar | EL |
| --- | --- | --- | --- | --- | --- | --- | --- | --- | --- | --- | --- | --- | --- |
| Young | 0.64±0.02^a^ | 8.1±0.73^a^ | 58.5±5.45^a^ | 2.3±0.14^a^ | 196.75±15.9^c^ | 47.81±1.9^c^ | 67.23±5.05^b^ | 279.81±10.31^c^ | 13.56±0^c^ | 2740±91.65^a^ | 0.93±0.05^a^ | 7.59±0.15^c^ | 0.39±0.08^b^ |
| Mature | 0.55±0.03^b^ | 6.38±0.33^b^ | 48.75±2.5^b^ | 1.93±0.06^b^ | 266.5±13.03^b^ | 36.11±2.24^b^ | 118.86±4.58^a^ | 410.78±30.93^b^ | 20.34±0^b^ | 1913.33±94.52^b^ | 0.91±0.04^a^ | 11.68±0.78^b^ | 0.47±0.03^b^ |
| Senescent | 0.44±0.05^c^ | 4.38±0.17^c^ | 37.75±6.08^c^ | 1.51±0.16^c^ | 306.5±15.42^a^ | 30.94±2.37^a^ | 127.83±7.93^a^ | 494.13±41.25^a^ | 29.38±3.91^a^ | 1773.33±94.52^b^ | 0.87±0.03^a^ | 16±2.92^a^ | 0.61±0.05^a^ |

Table S1 Physiological change of *Z. japonica* in responses to senescence

Note: Chl, Chlorophyll content; Pn, Net photosynthetic rate; Gs, Stomatal conductance; Tr, Transpiration rate; Ci, Intercellular-space CO_2_ concentration; IAA, IAA content; ABA, ABA content; APX, Ascorbate peroxidase activity; CAT, Catalase activity; POD, Peroxidase activity; RWC, Leaf relative water content; sugar, Soluble sugar content. EL, Electrolyte leakage; values are expressed as mean ± SD (n≥7). Different letters indicate significant differences at p ≤ 0.05 based on Fisher’s protected least significant difference (LSD) test.
